# Current Understanding of the Neural Stem Cell Niches

**DOI:** 10.3390/cells11193002

**Published:** 2022-09-26

**Authors:** Vicente Llorente, Pedro Velarde, Manuel Desco, María Victoria Gómez-Gaviro

**Affiliations:** 1 Instituto de Investigación Sanitaria Gregorio Marañón (IiSGM), Doctor Esquerdo 46, 28007 Madrid, Spain; 2 Departamento de Bioingeniería e Ingeniería Aeroespacial, Universidad Carlos III de Madrid, 28911 Leganés, Spain; 3 Centro de Investigación Biomédica en Red de Salud Mental (CIBERSAM), 28029 Madrid, Spain; 4 Centro de Investigaciones Cardiovasculares (CNIC), Melchor Fernández Almagro, 28029 Madrid, Spain

**Keywords:** brain, niche, stem cell, vasculature, neurogenesis, subventricular zone, subgranular zone

## Abstract

Neural stem cells (NSCs) are self-renewing, multipotent cells which give rise to all components of the central nervous system (CNS) during embryogenesis, but also activate in response to injury and disease and maintain a certain level of neurogenic activity throughout adulthood. This activity takes place in specialized regions of the brain, the neurovascular niches, whose main role is to control the behaviour of the CNS. In adult mammals, two main “canonical” niches have been described: The subventricular zone (SVZ) of the lateral ventricles and the subgranular zone (SGZ) of the dentate gyrus. This review discusses our current understanding of the neural stem cells and their canonical niches, as well as their structure, behaviours, and role in neural disease.

## 1. Introduction

The creation of new nervous system cells through the process of neurogenesis in mammals was originally thought to only take place throughout embryonic stages of development. However, this concept was questioned after the discovery of dividing cells in the hippocampus of adult rats [1], and of cells capable of in vivo proliferation and differentiation after extraction [2]. The fact that adult mammals are capable of maintaining neurogenesis throughout adult life thanks to multipotent neural stem cells (NSCs) is now widely accepted [3,4,5,6,7,8,9], and several functions and roles of neurogenesis and adult-born neurons have been described [10,11,12,13,14,15,16,17,18].

These NSCs reside in specialized niches in the adult mammalian brain, referred to as neurovascular niches (NVNs), including the subventricular zone (SVZ) and subgranular zone (SGZ). These niches are specialized microenvironments whose main purpose is to regulate self-renewal, proliferation, and differentiation of NSCs through their extensive interaction and participation with the niche itself, as well as maintain homeostasis and control central nervous system (CNS) behaviour [19,20,21].

## 2. Neural Stem Cell Niches: SVZ and SGZ

**Figure 1:**
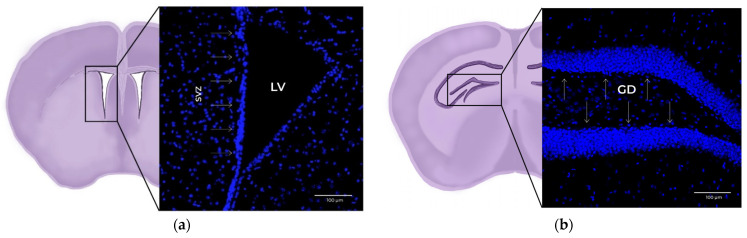
The 20x× magnification confocal microscopy images of coronal mouse brain slices. Blue: DAPI (cell nucleus marker). (**a**) Detail of the subventricular zone (SVZ) within the lateral ventricle (LV), marked with arrows. (**b**) Detail of the subgranular zone (SGZ) within the dentate gyrus (DG), marked with arrows.

Two NVNs in which neurogenesis takes place have been described in the adult mammalian brain: The SVZ, in the lateral ventricle (LV) [22,23], and the SGZ, located in the dentate gyrus (DG) of the hippocampus [4,24] (Figure 1).

### 2.1. SVZ

**Figure 2:**
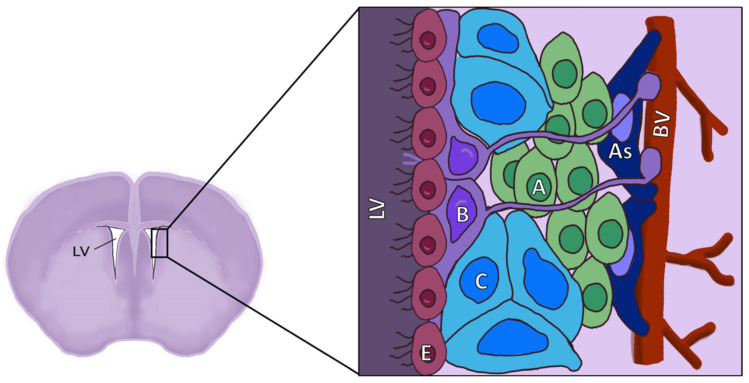
Diagram of the location and structure of the different cell types of the SVZ. LV: Lateral ventricle; E: Ependymal cells; B: Neural stem cells (NSCs), also known as radial glia-like cells (RGLs); C: Transient amplifying cells (TAPs), also known as intermediate progenitors (IPCs); A: Neuroblasts; As: Astrocytes; BV: Blood vessels.

The SVZ has been described as a site of neurogenesis in several mammalian species, including humans [22,25,26,27,28,29,30]. It is located in the lateral wall of the LV, and consists of different cell populations, including: NSCs, a monolayer of ependymal cells or E-type cells, transit amplifying cells (TAP or C-type cells, also known as intermediate progenitors or IPCs), neuroblasts (or A-type cells), astrocytes, and endothelial cells (BECs) that constitute the microvasculature [31,32] (Figure 2).

B-type cells follow cellular processes that, upon spread, lead to encountering cerebral capillaries and the ventricular wall, lined by the monolayer of E-type cells. This morphology suggests that B-type cells receive stimuli by direct contact from BECs and E-type cells. Despite being stem cells, B-type cells do not remain amorphous and undifferentiated, but rather showcase features that match those of differentiated astrocytes [33].

B-type cells retain the apical-basal polarity layout of radial glia (their predecessors), which contacts the ventricle and cerebrospinal fluid (CSF) that it contains through small, specialized apical processes with a single primary cilium. Moreover, up to a third of all cells in contact with the ventricular wall are B-type, and they are often found to be clustered [34]. This region of the SVZ is built in a “pinwheel” configuration, with the apical endings of B-type cells surrounded by E-type cells with large apical surfaces, and with intercellular junctions found between cells of the same type and both cell types [34].

In addition, these B-type cells can function as quiescent (qNSCs) or active (aNSCs). The aNSCs can transform into C-type cells, losing their GFAP expression and expressing neuronal differentiation transcription factor (ASCL1). Upon activation, their position shifts toward the vascular niche from the original ependymal location [35]. However, these movements are not unidirectional, and C-type cells can return and even interact with other NSCs. Moreover, dynamism is heavily age-dependent with morphological changes, and the tendency to stay in place increases alongside age [35].

B-type cell populations are not homogeneous, with differences present among cells of the matching activation state and in the diverse neuronal subtypes that they eventually give rise to [36]. This heterogeneity is mainly determined by the specific location within the niche itself, with B-type cells from the septal wall exhibiting a bias toward an oligodendrogenic fate, whereas lateral wall populations tend to result in neurogenesis [37]. Moreover, there are differences in gene expression profiles depending on whether cells originate from dorsal or ventral areas of the niche. These persist through the subsequent C- and A-type cell stages, delineating functional subclasses of adult-born new neurons as determined by the regional allocations of B-type cell populations they originate from [38].

Both B- and C-type cells are found near blood vessels, but most of the proliferative cells found in the SVZ are C-type; they undergo multiple rounds of mitosis to generate a sizable population before differentiating. In addition, those destined to be neurons give rise to A-type cells, which are also in close contact with blood vessels [31]. Most A-type cells leave the SVZ and migrate to the olfactory bulb (OB) through the rostral migratory stream (RMS), mirroring the flow of CSF [39] and moving longitudinally to vessels parallel to this RMS, suggesting that the capillaries function as migratory scaffolds [40]. Once in the olfactory bulb, they become mature interneurons [41].

However, this is not the only migration pattern, since a medial migratory stream (MMS) focused on the prefrontal cortex is also present in humans during early post-natal development stages. This stream is reduced in older children and nearly extinct in adults, and has yet to be identified in other vertebrates [42]. Moreover, children younger than 3 months of age showcase regions of high cell density adjacent to the anterior body of the lateral ventricle and within the neighbouring subcortical white matter, forming an “Arc” structure along sagittal sections. These are composed of migrating young neurons, which eventually move in chains or as individual cells toward the cortical tissue (including cingulate gyrus and prefrontal cortex) and differentiate into interneurons [43].

The “canonical” SVZ-RMS-OB migratory pathway may not be present in humans beyond immediate post-birth timeframes [42]. The human OB does show some remarkable plasticity [44,45,46], but this may be due to locally-generated neurons from resident NSCs or NSC-like populations [47], if it even takes place at all [48]. The RMS itself does appear to be present in humans, although lacking chain-migration [49] and with extremely low numbers of neuroblasts, which would render their sustained migration unlikely if not outright impossible due to the fact that neuroblasts are the ones that form and maintain the migratory route [22]. A possible ventricular extension conforming to a migratory path has been described [50], but this structure does not seem to be widespread among humans [51], even if there is some evidence of its existence [52]. A definitive answer to whether human olfactory bulbs are indeed recipients for newly generated SVZ neurons or they have purposes that are different from the rest of the mammals is yet to be determined, which might be relevant to discern how much of the knowledge on SVZ NSCs and niche obtained from animal models can be extrapolated to humans, if any at all.

### 2.2. SGZ

The SGZ is a germinal layer located between the densely packed granule neurons of the granule cell layer (GCL) and the hilus of the DG, where granule neurons are born, and neurogenesis takes place in close association with blood vessels [53,54,55]. Adult hippocampal neurogenesis has been observed in several mammals, including rodents, insectivores, carnivores, ungulates, and primates [56]. SGZ neurogenic activity begins relatively in early post-natal stages, and transitions to activity-dependent regulation after a re-structuring takes place to match the shift in function, hinting at an experience- and activity-based neurogenesis as opposed to be merely linked to adult development [57]. A high degree of synaptic formation and pruning within the hippocampus is paramount for memory encoding and processing, and this is dependent on two things: The immune system and the proximity of the cells to the vasculature [58]. Adult-born neurons originating from the DG are thought to partake in hippocampal-dependent cognitive development [59] and functions, such as spatial memory and pattern separation (the ability to discriminate between similar experiences) [60], non-destructive information encoding [61,62,63], inhibitory network regulation [64], or social memory maintenance (but not acquisition or retrieval) [65]. Moreover, a role in stress response and regulation through the suppression of the hypothalamic-pituitary-adrenal (HPA) axis has been described [16].

NSC populations are not identical between SVZ and SGZ, with small differences present in structure and location. Unlike their counterparts in the SVZ, SGZ NSCs are found deep in the brain parenchyma, surrounded by neurons and glial cells and away from the ventricular walls and the flow of the CSF [66]. SGZ NSCs seem to descend from a single precursor population in the dentate neuroepithelium (DNE), which expands and then migrates following the dentate migratory stream (DMS) to the primitive DG. These precursors acquire their adult properties during neonatal stages, but without any significant changes to their lineage specification, and maintain neurogenesis processes between peri- and post-natal stages [67,68].

**Figure 3:**
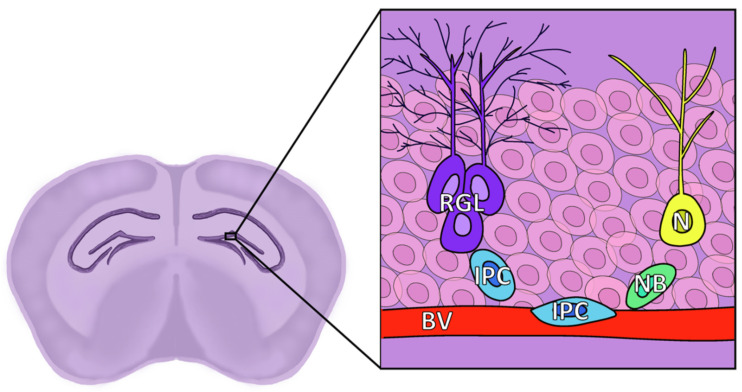
Diagram of the location and structure of the different cell types of the SGZ. RGL: Radial glia-like cells, corresponding to NSCs and known as Type 1; IPC: Intermediate progenitor cell, also known as Type 2; NB: Neuroblast, also known as Type 3; N: Granule neuron; BV: Blood vessel, granule cell layer represented by faint pink cells in the background.

Neural progenitors have been identified in the hippocampus as radial glia-like cells (RGLs) [69], also known as radial astrocytes [70] or Type 1 cells [71]. Similar to the B-type cells of the SVZ, they express GFAP, nestin, and Sox2, and possess radial processes that contact vessel endothelial cells in specialized irregular areas of the basement membrane, providing access to blood-borne substances despite the existence of the blood-brain barrier (BBB) [72]. Intermediate progenitors, also known as Type 2 cells, are equivalent to C-type cells from the SVZ, are highly proliferative, and normally express nestin and DCX. These eventually transform into neuroblasts, also known as Type 3 cells, which express DCX and PSA-NCAM, but not nestin. They migrate along the blood vessels until they reach the granular layer, where they differentiate into mature dentate granular neurons [54] (Figure 3).

The extent to which hippocampal neurogenesis persists into adulthood in humans is not yet clear, as it is a controversial topic with seemingly contradictory results. Certain studies have found no significant levels of neurogenesis beyond early childhood [73,74,75], while others point at lifelong neurogenic activity [3,5,9,76,77,78], with the issue far from settled [79,80,81]. The scale seems to tip in favour of adult hippocampal neurogenesis, with indirect (functional) evidence pointing toward it as well, but many questions remain unanswered [82]. Considering the amount of research carried out on the topic of human hippocampal neurogenesis and the fact that the bulk of the functional evidence of adult neurogenesis that is relevant to neurological processes is aimed at this niche, the SGZ is the most likely out of the two canonical niches to be the first to have its secrets unravelled in humans. However, that unravelling has not been reached yet. 

## 3. Niche Components

The neural stem cell niches are formed by various cell types, such as BECs, pericytes, astrocytes, and microglia. The proper function of cerebral microvascular networks as well as the niche itself depends on the synergistic interaction between them [83,84,85].

Pericytes are mural cells that directly adhere to capillaries and regulate microvessel permeability, vasoconstriction, and BBB-specific gene expression [84,86]. SVZ NSCs control the capillary tone and blood flow in the surrounding area through pericyte activation [87]. Astrocytes are glial cells of the CNS which are commonly identified through GFAP expression [88]. Microglia are mononuclear phagocytes, and represent the main permanent immune cell population in the CNS [89]. In addition, the SVZ-specific population showcases a unique phenotype and transcriptome when compared to other regions of the brain [90,91]. SVZ/RMS microglia present a reduced phagocytic activity, and instead take part in the migration of SVZ-generated neurons toward their final destination in the OB. The disruption of these populations has adverse effects on neuron migration and survival [90]. Moreover, SVZ microglia are capable of acting as a hotbed for repopulation following whole-brain microglia depletion, and are resistant to the inhibitors [92]. 

Other key components of the niche include the vasculature, the extracellular matrix, and the CSF, as well as the lateral ventricle choroid plexuses (LVCPs), which are primary producers of CSF and source of secreted factors with different effects on SVZ NSCs and their progeny [93].

**Figure 4:**
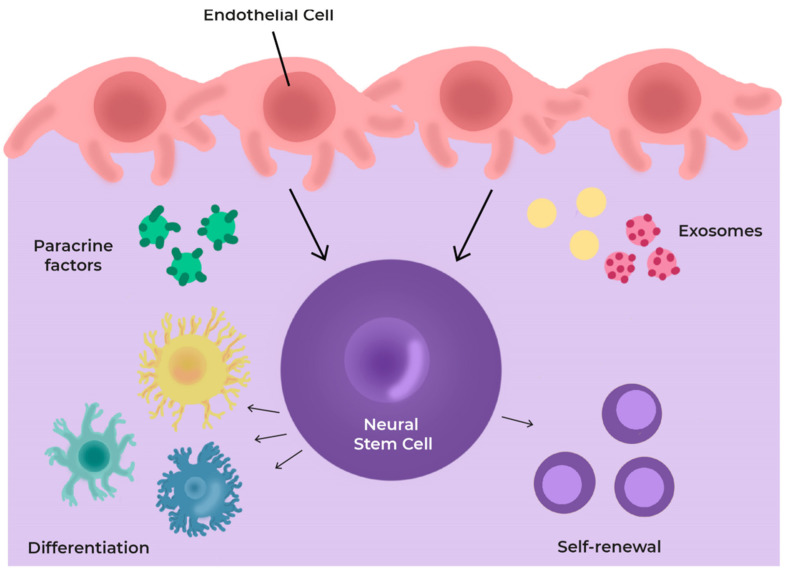
Scheme of cells and secretome in the neurovascular niche (NVN). The niche components regulate cell differentiation through the production and release of several paracrine factors and exosomes.

All these components determine the behaviour and fate of NSCs through the production and release of a number of paracrine factors, directly or contained in exosomes. They decide how quickly NSCs divide and the type of cells they give rise to [94,95,96] (Figure 4).

### 3.1. Vasculature

**Figure 5:**
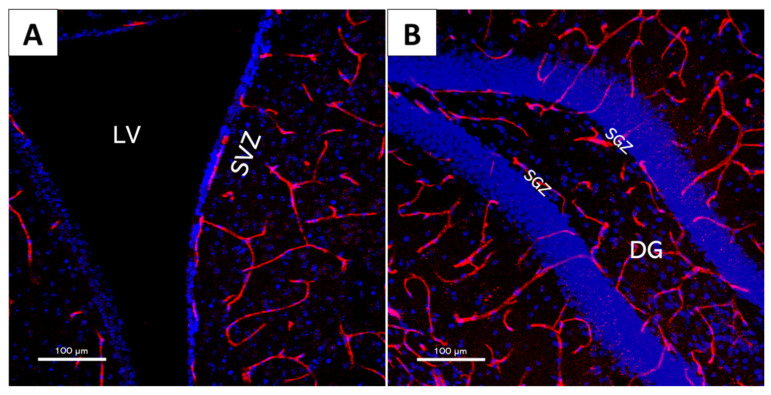
The 20× magnification confocal microscopy images of mouse brain slices showcasing neural stem cell niche structure and their intricate vascular network. Red: CD31 (vascular endothelial cell marker); Blue: DAPI (cell nucleus marker). (**A**) Lateral ventricle (LV) and subventricular zone (SVZ); (**B**) dentate gyrus (DG) and subgranular zone (SGZ).

The distinctive requirements of the adult mammalian brain result in the development of unique vascular structures. In the CNS, BECs form the highly restrictive and semi-permeable BBB, which separates circulating blood from the cerebral extracellular space and strictly regulates the transport of molecules to maintain a highly specific and controlled microenvironment [97]. The majority of proliferative NSCs and NPCs are commonly found near specialized blood vessels [53] (Figure 5), with BECs playing a key role in regulating the behaviour of these cells by, for example, maintaining the quiescence of NSCs [98]. 

This permeability extends to the SVZ, with vascular endothelial cells lacking astrocyte end feet and pericyte coverage. On the other hand, the SGZ does not present it under normal homeostatic conditions. Therefore, the SVZ allows for direct interactions between NSCs and vascular endothelial cells, which play a crucial role in NSC regulation of quiescence and differentiation. For example, the laminin-binding α6β1 integrin receptor (expressed in NSCs, but lost after differentiation) maintains NSC adhesion to blood vessels, while the disruption of its function results in NSCs moving away from the vascular network and entering a proliferative state [31]. The homing mechanism that directs NSCs toward blood vessels in the first place and upregulates α6β1 integrin expression to favour this adhesion involves interactions between stromal-derived factor 1 (SDF1) mainly produced by ependymal cells and CXC chemokine receptor 4 (CXCR4) expressed in NSCs [99]. Similarly, quiescent NSCs are maintained in that state through cell–cell interactions with BECs involving the ephrinB2 and jagged1 transmembrane ligands, among other cues. These suppress qNSC receptivity to proliferation and differentiation-inducing factors and enforce stemness [98].

Furthermore, vascular endothelial cells communicate with NSC through secreted factors (such as PEDF and betacellulin, which promote self-renewal and proliferation, respectively [19,100]), as well as NSC and endothelial cell expressed factors (such as VEGF and ang-1).

### 3.2. Neural Stem Cells

NSCs are defined as cells that can self-renew (i.e., proliferate generating identical cells) and that are multipotent (they can give rise to the major neuronal lineages, including neurons, astrocytes, and/or oligodendrocytes). During embryogenesis, NSCs are localized in the ventricular zone of the neural tube and can give rise to all of the necessary cell types for CNS formation [101]. This includes the glial lineage (astrocytes and oligodendrocytes) and the neuronal lineage (granular and periglomerular neurons) [2,102]. Moreover, they present activity in response to injury, resulting in increased proliferation of new neurons and possess a protective role [103,104,105].

As with many other proliferative cells, a vast majority of NSCs remain quiescent, in a state of reversible cell cycle arrest that prevents proliferation, thus retaining their future proliferation potential and avoiding damage accumulation that may hinder that purpose. Certain NSCs remain the same from early stages of embryonic development. They only become activated significantly later at post-natal or even adult stages and continue to produce neurons [106]. Quiescent NSCs display different cell cycle properties, behaviours, and molecular signatures from their activated (actively generating differentiated progeny) states, and give rise to different final cell populations: Neurons and colony-forming cells in the case of the latter, and olfactory bulb interneurons in the case of the former, with the result of quiescent NSCs showcasing significantly slower kinetics and little- to no-colony-forming abilities [107]. Moreover, activated SVZ NSCs possess increased levels of epidermal growth factor receptor (EGFP), which controls some of their defining characteristics compared to their quiescent counterparts [108,109].

Division of active NSCs does not follow a fixed mode, with two distinct possibilities: Asymmetric division, to produce one differentiated cell and one stem cell, and symmetric division, to give rise to two differentiated cells or expand the NSC population. In the case of symmetric division, asymmetry is still required for a proper function, but is achieved on a population level through a balance on the rate of expansion and differentiation [110,111]. SVZ NSCs tend to follow the latter, with roughly 80% dividing into differentiated cells, while 20% are tasked with self-renewal. This layout results in the progressive depletion of NSC “reserves”, but allows for a large number of generated progeny for the OB and decouples NSC proliferation from differentiation [112]. On the other hand, SGZ NSCs mainly follow an asymmetric division, resulting in the maintenance of the stem cell population while producing a differentiated cell at the same time. Symmetric differentiation and self-renewal take place as well, but at a more reduced frequency [69,113].

Some NSCs return to a quasi-quiescent state after becoming activated and going through a few divisions, although this state does not last in the long term, and they soon return to an active state [113]. This would suggest that previously active NSCs are more likely to become activated again than ones that have been quiescent for a long period of time [114].

### 3.3. SOX

The Sox proteins are a group of transcription factors that share a high-mobility-group (HMG) DNA-binding domain originally identified in the mammalian sex-determining region Y protein (SRY). These proteins are highly conserved through evolution, and show a very restricted and targeted binding behaviour [115,116]. While the group comprises several members, varying levels of sequence identity between them result in the formation of subgroups. These display different functions from each other, while in-group members have overlapping functions due to identity. This, combined with a high molecular versatility and target gene selectivity, allows the same factors to play different roles in different tissues and processes [117]. Sox proteins primarily act alongside “partner” factors to carry out their actions (with the binding of a single partner-less Sox protein to a DNA site having no effect), although their function can be further regulated by their concentration, by post-transcriptional modifications by miRNAs, by interactions with other proteins, or by auto-regulation of their expression by other members of the Sox family [118]. Sox group members are found in several developmental processes [118]. One example is in the neural crest, a cell population that gives rise to most of the craniofacial complex, melanocytes, the peripheral nervous system, and other facial structures [119].

The SoxD subgroup is composed of three genes: Sox5, Sox6, and Sox13. These are among the largest members of the Sox family and present a high level of sequence identity, but only share the HMG itself with other Sox members. SoxD genes are expressed in diverse cell types throughout development and adulthood, most notably in critical aspects of development, such as cell fate determination [120]. During development, all three factors are expressed in CNS cells and cartilage tissues, with unique expression shown in melanocytes and Th17 cells for Sox5 [121,122], myocytes and erythroid cells for Sox6 [123], and neuroepithelium, arterial walls, and T lymphocytes for Sox13 [124,125,126]. SoxD members are involved in corticogenesis, with Sox5 regulating differentiation for certain sets of cortical neurons and showcasing a mutually exclusive expression pattern with Sox6 [127,128]. Sox13 is co-expressed alongside Sox5 and Sox6 in oligodendroglial-lineage cells, and complements their development [129].

In the context of the NSC niches, Sox5 and Sox6 are predominantly expressed in SGZ NSCs, with most showcasing co-expression of both factors alongside Sox2, and with Sox5 expression augmented in aNSCs compared to qNSCs. In turn, an increase in Sox5 expression results in hindering of their ability to enter a quiescent state when prompted by other markers, such as bone morphogenetic protein-4 (BMP-4). The absence of Sox5 and Sox6 in SGZ NSCs results in their inability to become activated, pointing to both factors as crucial for qNSC activation [130].

Sox2 belongs to the SoxB1 subgroup, and has a high functional connection to stem cell status. In mammals, it is expressed and required from early stages of embryonic development, both in NSCs of the blastocyst inner cell mass and in neural epithelial cells [131]. At later stages of development, Sox2 is preferentially maintained in stem cells of different tissues, most notably the developing and post-natal nervous system [131]. Within the nervous system, Sox2 is highly expressed in NSCs and NPCs, constituting the ventricular zone of the developing neural tube, and its conditional deletion impacts the function of these NSCs both in vitro and in vivo. Studies have shown that it is absolutely necessary for the development of the mouse olfactory neuroepithelium, a neurogenic epithelium in which long-term stem cell self-renewal occurs and which, in addition, can reprogram differentiated cells to induce pluripotent stem cells (iPSCs) [131].

Sox9 is another Sox-family transcription factor that partakes in nervous system development, which is required for initial neural crest induction and gliogenesis [132]. It is required to maintain NSC multipotentiality in the adult brain, with its absence resulting in a lack of astrocytes, oligodendrocyte, and neuron generation [133]. Sox9 downregulation is required for neurogenesis, while its overexpression results in maintenance of SVZ cells as GFAP+ astrocytes with no neuronal production. This downregulation is carried out by miR-124, which then regulates the progenitor count and timing of differentiation and maintains homeostasis in the niche [134,135].

Sox4 and Sox11 belong to the SoxC subgroup, which regulates mammal nervous system development. Both factors are critical for the proper generation of sympathetic ganglia and spinal cord, among other tissues [136,137]. SoxC group members promote the expression of differentiated neuronal proteins, with Sox11 as a promoter of neuronal-specific gene expression [138]. Sox4 and Sox11 expression in DG-lineage begins after neuronal fate commitment takes place, as they regulate the initiation of maintenance of the neurogenic process and their absence results in hindrance of neuron production [139].

### 3.4. Other NSC Markers

Doublecortin (DCX) is a microtubule-associated phosphoprotein expressed in neuronal precursors of the developing CNS and required for neuronal migration and differentiation [140]. DCX influences stabilization and destabilization of MTs, orchestrates cellular dynamics, and links cytoskeletal components, which is unique among other microtubule-associated proteins in collectively carrying out all three of these functions [141]. DCX expression in the adult brain is mainly restricted to neurogenic niches, i.e., SGZ and SVZ [142], and, in particular, to proliferating progenitors and immature neurons, which is absent at any point before or after those stages [143].

Neuroepithelial stem cell protein (nestin) is a class VI intermediate filament protein that makes up a critical component of the cytoskeleton. It is present during CNS development and in adult NSCs, and its expression is downregulated after differentiation takes place [144]. The role played by the cytoskeleton in the process of cell division and differentiation gives nestin a relevant profile in stem cells, alongside other paths of action, such as a regulatory effect on assembly and disassembly of other cytoskeletal components, as well as factors of differentiation [144]. In the adult brain, nestin positive cells correspond to NSCs and progenitor cells in the SVZ, RMS (where progressively reduced expression patterns are found, corresponding to migrating only-differentiated neurons) and SGZ, and serve as accurate markers for these cell types [145]. Although immature neurons are also capable of showcasing reduced nestin expression, expression levels are many times lower than those of progenitor cells, thus still maintaining its reliability as a marker [146].

Polycomb repressive complex 2 (PRC2) mediates gene silencing that results in tissue maintenance and developmental process control. It consists of several different subunits, including the core protein embryonic ectoderm development (Eed). Eed is extensively expressed on cells belonging to the SVZ lineage, and its loss reduces NSC proliferation and neurogenesis in the SVZ through an increase in the production of the transcription factor Gata6. Gata6, in turn, maintains cyclin-dependent kinase inhibitor 1 (p21) [147].

Oligodendrocyte transcription factor (OLIG2) is a basic helix-loop-helix (bHLH) transcription factor that determines oligodendrocyte lineage by controlling oligodendrocyte precursor cell (OPC) specification, differentiation, and myelination [148]. It is highly expressed in undifferentiated and proliferative NSCs, where it regulates proliferation and morphology [149]. Its export from the nucleus is critical for astrocyte differentiation. Therefore, it is located in the nucleus of NSCs, while astrocytes showcase cytoplasmic expression [150].

Lunatic fringe (Lfng) is a fringe protein involved in the regulation of Notch signalling, and it appears to be selectively expressed in adult NSCs, possibly as a method of communication between NSCs and their differentiated “daughter” cells to properly regulate cycling and maintain enough stock to maintain a steady progeny. Lfng expression is restricted to the SGZ and can act as a marker for hippocampal NSCs [151].

Akhirin (AKH) is a new soluble molecule identified recently as a stem cell maintenance regulator. The potential role of AKH regarding neurogenesis is an important novel discovery. AKH may be involved in differentiation, proliferation, and self-renewal process in NSCs/NPCs located in both niches. Similarly, the lack of akhirin is observed to produce limited growth and development in these neurogenic regions [152,153].

BASP1 is considered a signal processing protein that plays critical roles in synaptic plasticity and neurite outgrowth and belongs to a family of growth-associated proteins. While expressed throughout the whole brain during development, it is restricted to neurogenic regions in the adult brain, and acts as a marker for NSCs, with robust expression found on NSCs of the SGZ and SVZ [154].

### 3.5. Extracellular Vesicles

With communication between cellular components as significantly critical for the proper maintenance of neurogenic niches, the mechanisms of this communication are key to understanding these processes. Extracellular vesicles (EVs), among which exosomes are found, are a novel pathway which appears to play a relevant role in stem cell niches. EVs transport proteins, lipids, and nucleic acids. In particular, they are rich in small non-coding RNAs (ncRNAs), the most common being miRNAs, thus altering the target cell’s processes, phenotype, or physiology [155]. Exosomes are formed in a separate way from other vesicles and can stimulate receptors on target cells, or transfer superficial receptors across different cells. Moreover, they are capable of penetrating the blood-brain barrier (BBB) [156], as well as inducing the disintegration and transmigration of other cells across it [157].

**Figure 6:**
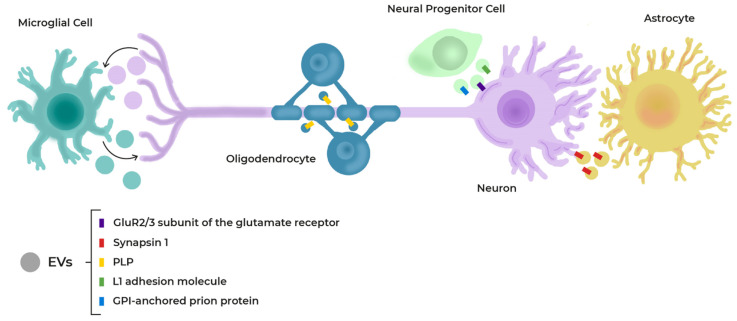
Overview of potential extracellular vesicle (EV)-mediated cellular interactions in the nervous system, and the receptors or molecules involved in their integration.

EVs are released by all types of cells in the brain: Neurons, astrocytes, oligodendrocytes, microglia, and neural progenitor cells [158]. Among other processes, exosomes take part in bidirectional oligodendrocyte-neuron communication [159]. The majority of the cell types present in the niches secrete or are targets for EVs, including exosomes, and these are also relevant in communication among neural cells and with blood (Figure 6).

Human umbilical vein endothelial cell (HUVEC)-derived exosomes have been shown to increase proliferation, decrease apoptosis, and maintain multipotency in NSCs [160]. Multipluripotent mesenchymal stromal cell (MSC)-derived exosomes can significantly increase neurogenesis in the DG [161]. In addition, NSCs can propagate some of their immune modulatory activities and activate Stat1 signalling on target cells through EV secretion [162]. NSC-derived EVs can create a positive neuroprotective and regenerative effect on ischemic stroke and traumatic brain injury models [163,164].

Micro RNAs (miRNAs) are small noncoding sequences of RNA between 18–25 nucleotides in length that regulate gene expression at the translation level [165]. They take part in developmental and disease processes, and act as repressors by guiding the cleavage of target miRNAs, or by directly inhibiting their translation [166]. The miR-124 is a heavily conserved miRNA whose variants can account for 25% to 50% of all brain miRNAs [167].

Several steps of adult neurogenic processes are regulated by miRNAs, including proliferation, differentiation, glial cell proliferation/differentiation and angiogenesis, neuroblast migration and neuron maturation, and neuronal fate, etc. While the specific transport and uptake mechanism has yet to be determined, all the relevant miRNAs have been found in exosomes, sometimes very extensively, thus presenting a strong case for exosome-mediated communication taking place in this context [155].

Modulation of cell aging is carried out by hypothalamic NSC partly through the release of exosomes carrying miRNAs [168]. In addition, embryonic NSC-derived exosomes facilitate the differentiation of NSCs and the maturation of both neuronal and glial cells. This is carried out through the delivery of miR-9, which is abundantly expressed and is a key regulator of neurogenesis timing [169]. Microglia and NSC carry out exosome-mediated crosstalk as part of the injury repair process, and this is carried out through the exchange in miRNAs focused mostly on proliferation [170]. Neonatal NSCs from the SVZ release extracellular vesicles targeted to microglia, which regulate their physiology and morphology [171].

The miR-204 maintains a state of readiness in qNSCs, ensuring their ability to rapidly become activated and differentiate while stopping the initiation of this response. This priming is carried out by the choroid plexus through secretion of this miRNA, and regulates neurogenic fate determinants, such as Sox11, Meis2, or Pax6 [172].

### 3.6. Extracellular Matrix

The extracellular matrix (ECM) of the niches also plays a role in the regulation and maintenance of NSCs. Standard brain parenchyma does not easily allow for neurogenesis, or the integration of new neurons to take place other than in cases of injury, as opposed to niche ECM [173,174]. In the SVZ, the ECM takes the form of “fractones”, complex structures displaying a fractal ultrastructure (thus the name) that consist of collagen IV, laminin, nidogen, and heparan sulphate proteoglycans (HSPGs) and are affiliated with basement membranes despite their distinctive morphology [175]. 

These structures act on niche cells through their interactions with growth factors and cytokines, diffusible signal proteins that regulate cell dynamics both near the production site (paracrine action) and at longer distances through diffusion in the intracellular space, or through the bloodstream akin to hormones [176]. Moreover, physical characteristics are relevant as niche ECM showcases a significantly higher level of stiffness compared to standard brain parenchyma, and neurogenesis itself is reactive to differences in its value; higher levels of stiffness seem to correlate with an increase in neurogenic activity [177,178].

Several of these signal molecules bind to heparin, with HSPGs in the ECM facilitating binding to their corresponding receptors in target cells [179]. Fractone-bound HSPGs in the SVZ interact with fibroblast growth factor-2 (FGF-2) [180], bone morphogenetic protein-7 (BMP-7) [181], and bone morphogenetic protein-4 (BMP-4) [182], sequestering and regulating their action in altering cell proliferation behaviour. Moreover, fractones include an extra component in the form of bulbs, small spherical deposits found at the ependymal layer, and at the centre of pinwheels. These bulbs are produced by ependymal cells and take part in NSC regulation through their laminin composition, with the absence of the latter resulting in increased cell proliferation and reduced amount of qNSCs [183].

The expression of the ECM cross-linking protein transglutaminase 2 (Tgm2) is enriched in SVZ ECM and is also expressed in ependymal cells and NSCs in contact with the ventricle. It promotes neurogenesis and NSC proliferation in vitro and in vivo through intracellular functions, although as a multifunctional enzyme it may also carry out extracellular functions in vivo, and it contributes to ECM stiffness, potentially further partaking in regulatory activity [178].

In the case of the SGZ, β1-integrin is part of the family of transmembrane receptors that mediate cell-extracellular matrix and cell–cell interaction and is highly expressed in NSCs where it promotes and regulates maintenance. β1-integrin is critical for the maintenance of the structural integrity of DG, inhibition of astrocyte differentiation, maintenance of NSC populations, and regulation of neurogenesis. Ablation of β1-integrin results in reduced neurogenesis as well as depletion of the radial NSC population, with these effects showing sex-specific differences in time-course [184].

### 3.7. Cerebrospinal Fluid

The cerebrospinal fluid constitutes the environment of the brain and spinal cord, and thus envelopes and protects the CNS. It is secreted by the choroid plexus in the ventricles, and primarily flows through cisterns and the subarachnoid space until its absorption by the arachnoid villi and drainage into the blood or extracranial lymphatic system [185].

CSF is not simply a protecting layer, as it also maintains homeostasis of the brain fluids (as an ionic buffer) and provides the means for waste disposal. Inadequate amounts of CSF can result in impairment of brain development or other pathologies, such as hydrocephaly [186]. Moreover, CSF partakes in component exchange with the interstitial fluid and mediation of several signalling pathways. Some of these pathways involve B-type cells from the SVZ, which are in direct contact with the CSF itself through primary cilia [187]. A wide range of proteins, neurotransmitters, and peptides are present in the CSF, and are secreted by neurons, glia, and ependyma, transported through specialized mechanisms, or originate from cells present in the CSF itself; with all varying in composition as the flow moves throughout its course [188]. Ependymal flow of CSF creates a chemorepulsive gradient throughout the CSF flow, which matches movements from the RMS followed by SVZ-originated neuroblasts, and guides and supports the migration of the latter [39]. 

CSF is a key factor in embryonic brain growth, morphogenesis, and histogenesis through its interplay with the neuroepithelium (primary neural stem cells in early stages of development) [189], and it is involved in the synthesis and transport of neurogenic factors [190]. Embryonic CSF holds a “cocktail” of growth factors and morphogens capable of inducing replication and neuronal differentiation in NSCs. This effect can be induced in adult NSC populations and results in increased neural progenitor and neurogenesis activity. In addition, proper maturity of the newly-generated neurons after migration is carried out [191]. Adult CSF can still trigger a differentiative response in NSCs *in vitro*, although the specifics are not clear. One study found the response to be biased toward astroglial fate in detriment of NSC proliferation and maintenance or a neural differentiation fate [192], while another study resulted in an increase in proliferation, restriction in motility, and differentiation with neural fate propensity [193]. The effect of CSF on neural progenitors takes place, among other factors, through the delivery of insulin-like growth factor (Igf) signalling, the regulation of which extends to disease states, as well [194].

Another key system in CSF regulation of SVZ NSCs is the epithelial sodium channel (ENaC), which is a voltage- and ligand-independent ion channel sensitive to fluid shear stress expressed in kidneys and lungs (including stem cells), as well as several locations in the nervous system, including brain centres controlling fluid volume or blood pressure. ENaC is differentially expressed in SVZ NSCs and progenitors and is critical for their proliferation, with increases in CSF fluid flow triggering fast proliferation responses [195]. While it is suggested that this mechanism is used to detect the mere presence of the ventricle (with fluid flow representing CSF and thus the location of the ventricle itself), it is also likely to be a way to detect changes in physiological and pathological conditions of the brain, as these in turn trigger changes in CSF flow and composition [196].

## 4. Neural Stem Cell Niches and Disease

One of the main motivations to enhance the study of the brain at a cellular level is fighting the consequences of neurodegenerative diseases and medical conditions. These health disorders directly result in cell death, causing structural damage that results in dysfunction of nervous activity and cognitive impairment. Neurodegeneration is a process that can appear in various levels of failure of the neuronal activity extending from molecular to systemic [13]. 

In the SVZ of rodents, suppression of the olfactory neurogenic pathway translates into deficiencies in mating, breeding, and integral abilities to the survival of most mammals [18]. On the other hand, the DG restraint of adult neurogenesis results in essential alterations in learning and memory [197]. Alterations in DG neurogenic activity and homeostasis might be relevant factors of hippocampal disruption and the ensuing cognitive and degenerative effects of pathological processes, such as amyotrophic lateral sclerosis (AHN), Huntington’s disease (HD), Parkinson’s disease (PD), dementia with Lewy bodies, and frontotemporal dementia [198]. Disruption in the SVZ and DG has been associated with PD, HD, Alzheimer’s disease (AD), and epilepsy [199,200,201]. Therefore, understanding the mechanisms of neurogenesis regulation can prove crucial for finding robust solutions for these illnesses.

### 4.1. Aging

Aging has been established as the most significant risk factor for neurodegenerative diseases. Suffering from a neurodegenerative disease, such as PD, amyotrophic lateral sclerosis (ALS), HD, or dementia hastens aging of the brain, in relation to disorders in NSC niches [202]. 

During aging, NSCs and their progenitors exhibit reduced proliferation and neuron production [203,204], which is thought to contribute to age-related cognitive impairment and reduced plasticity that is necessary for some types of brain repairment [205]. System-wide blood-borne factors whose levels increase with age in healthy individuals take part in this process, and the exposure of young individuals to the old-age-mimicking levels of these factors results in similar impairments and reductions in neurogenic activity [206,207]. Moreover, the opposite applies and the exposure of aged individuals to blood-borne factors and concentrations associated with earlier life stages result in vascular remodelling and increased neurogenic activity [208,209]. Some of these processes involve BEC activity, triggered by the action of the aforementioned factors in the systemic circulation [209,210].

The structure of the niche vascular network itself also changes with age, with decreased vessel diameter and increased tortuosity showcased by older individuals [211]. These parameters can result in reduced blood flow and even ischemia [212], and are a departure from the original values of the SVZ, where tortuosity is lowest close to the ventricle and ependymal cell layer [213]. However, these changes are not the same between males and females, with females showing decreases in vessel density and tortuosity while diameters increase. Moreover, females display a lower amount of age-related neurogenesis disruption and progenitor proliferation [211].

Furthermore, microglia take part in hippocampal neurogenesis regulation through phagocytosis of cells entering apoptosis between the progenitor and neuroblast stages, thus maintaining homeostasis [214]. The presence of microglia brandishing the phagocytic pouches required for this effect is reduced in aged individuals, pointing at an age-induced disruption in microglial function as another factor in age-induced alterations in neurogenic and cognitive functions [198].

Another contributor to the increase in speed in the aging process is mitochondria. Mitochondria are crucial regulators of cell death, and thus intrinsically concerning in neurodegenerative diseases. This contribution is made through the accumulation of mitochondrial DNA (mtDNA) mutations and the resulting net production of reactive oxygen species (ROS) [215].

### 4.2. Stress

Stress is considered another risk factor for neurodegeneration. PD has been associated with post-traumatic stress disorder (PTSD) [216]. Some studies show that prolonged-lasting midlife stress is correlated later in life with the increase in CSF biomarkers considered to be characteristic of unspecific neurodegenerative processes [217]. SGZ neurogenesis takes part in the stress response by acting as a “stress buffer” and reducing its magnitude. Conversely, adrenal glucocorticoids (GDs) inhibit neurogenic activity [16,218]. The neurogenesis-suppressing effect of GDs and their negative impact on SGZ cells may take place through autophagic processes, where cytoplasmic components are sequestered in double-membraned autophagosomes for lysosomal degradation, as opposed to conventional apoptotic activity [219]. The anti-depressant effect carried out by ketamine takes place through the increase in activity of adult-born immature granule neurons in the SGZ via α-amino-3-hydroxy-5-methyl-4-isoxazole-propionic acid receptor (AMPAR) activation, and this activation alone is enough to induce beneficial effects [220].

### 4.3. Stroke

When blood supply to a region of the brain is interrupted, the affected tissue receives an insufficient level of oxygen, which in turn causes cell death. Although not broadly recognized as a neurodegenerative disease, there are plenty of studies reporting stroke-induced secondary neurodegeneration and glial disturbance, showcasing accumulation of neurotoxic proteins, such as amyloid-β in a similar way to other neurodegenerative diseases [221]. Another result of the injury is the generation of a hypoxic environment, which in turn causes an increased rate of differentiation in NSCs compared to standard conditions, providing a neurogenic response. Nevertheless, there are limits to this response, as determined by the minimum oxygen levels that allow proper NSC differentiation [222]. Transplantation of NSCs into the stroke site reduces lesion size and apoptosis rate in the penumbra throughout their secretion of neuroprotective paracrine factors, although the elevated rate of cell death on the graft caused by the hostile environment hinders these efforts [223]. Nuclear factor-erythroid 2-related factor 2 (Nfrf2) takes part in the maintenance of homeostasis by enhancing the production and release of antioxidant, anti-inflammatory compounds, and the induction of an increased expression enhances NSC survival on the injury site, and thus, the neuroregenerative effect these partake in [224].

### 4.4. Alzheimer’s Disease

AD is the most common neurodegenerative disorder, although most of its causes are still unknown. Neurogenesis impairments have been linked with AD-induced abnormalities of the brain fatty acid metabolism. An accumulation of triglycerides and building of mono-unsaturated fatty acids were observed in the NSC forebrain niche, with the loss of NSC activity possibly reversed or decreased through inhibition of the transformation of saturated to mono-saturated fatty acids in the brain. Moreover, a relation was established between qNSC sensitive to certain levels of fatty acids at local level [225]. Analysis of vasculature permeability in aging and mild cognitive impairment patients revealed that progressive BBB breakdown begins in the hippocampus and may contribute to early stages of dementia associated with AD [226]. Fibrinogen is proposed as a regulator of NSPC-derived astrogenesis from the SVZ through the BMP receptor signalling pathway [227].

### 4.5. Parkinson’s Disease

PD is associated with functional changes suffered by microglia and astrocytes in the *substantia nigra*, resulting in decreased dopamine levels due to the loss of midbrain dopaminergic neuron populations [228]. The therapeutic use of NSC grafts has been demonstrated to drive an astrocyte-dependent Wnt1 signalling activation, which in turn favours the neurorestorative activity of the midbrain dopaminergic neurons [229]. Adult SVZ and SGZ have been studied to pursue a neural and glial cell replacement. Diseases in which only one or few cell types are lost might increase the chances of success in cell replacement strategies via NSCs treatment. ES cell-derived and adult NSCs are capable of induction to generate oligodendrocyte precursors [230,231]. A novel model of NSC-ES coculture combined to produce a similar environment of the neurovascular niche, showcased that the proper selection of NSCs combined with midbrain dopaminergic neurons that integrate into the existing nigrostriatal DA pathway could result in a drastic reduction in the symptoms of PD [232].

### 4.6. Glioblastoma

Adult glioblastoma (GBM) is the most common type of primary malignant brain tumour and the most aggressive one [233]. Despite multiple advances in therapies and care, it still displays a very poor prognosis in most cases, with low long-term survival rates, and a particularly negative effect on patients and caregivers due to the associated decline in quality of life and neurological function [234].

Several studies have established a relationship between contact or closeness of the tumour with the SVZ and a poor prognosis and lower survival rate, as well as a potential cause of GBM recurrence [235,236,237,238]. SVZ irradiation may even present itself as a potential treatment to improve prognosis and increase survival rates [239,240].

While originally thought to be exclusively generated from glial cells, further research has pointed at multiple cell types with stem-cell-like properties as the source. These cancer stem cells (CSCs), or rather glioma/glioblastoma stem cells (GSCs) can differentiate and self-renew, and also provide chemotherapy and radiotherapy resistance [241]. GSCs, in turn, would have been generated from NSCs, and SVZ NSCs in particular, after an oncogenic transformation [242]. GSCs share many properties and markers also present in NSCs. Similarly, astrocyte-like SVZ NSCs can acquire GBM driver mutations and migrate away from the SVZ, resulting in uncontrolled proliferation and high-grade malignant glioblastomas in distal regions [243,244].

## 5. Current Limitations

The issue of unravelling the details of adult neurogenesis is made even more complex by the fact that neurogenic processes show great variation between species, and are also affected by age and pathological states, potentially rendering any results difficult or outright impossible to extrapolate between them [245]. Moreover, these differences may be related to the actual role and relevance that adult neurogenesis has for each of them, if any at all, which would thus result in a fundamental incompatibility between the dynamics of two species, unless their “need” for neurogenic processes happen to align (and even then, extrapolation might not prove successful) [246].

Even the size of the brain itself could influence neurogenesis, with larger brains making it significantly harder for new neurons to migrate through the distances between niches and target areas, and thus, rendering them ineffective. On the other hand, this could be circumvented through mechanisms that bypass these restrictions [247]. For example, the maintenance of local progenitor populations in distant areas from the niches (such as those suggested as a source of non-canonical neurogenesis) could prevent the need for young neurons to migrate long distances by virtue of simply being located significantly closer to their target regions; therefore, this generates different requirements for dynamics and cell populations involved in these processes.

Another issue concerns the actual methods used to detect neurogenesis itself, which are not without their shortcomings. Markers, such as DCX, may accurately label cells with characteristics matching those of immature neurons, but they do not necessarily imply that these immature neurons were newly generated in adulthood as part of active neurogenic processes. Several regions of the brain can hold resident populations of immature neurons that originated during embryogenesis, which can remain developmentally frozen for long periods of time (up to decades) and which provide a non-proliferative source of plasticity throughout adulthood [248,249,250,251,252]. These carry out “protracted” neurogenesis processes as an extension of embryonic development, in contrast with “persistent” neurogenesis which remains throughout aging, even with reductions in activity [253].

Existing mature neurons may also be contributing to this confusion, as certain conditions can induce the expression of cell cycle markers [254,255], and the possibility of undergoing a “dematuration” process, which results in gene expression patterns matching those of immature neurons [256,257]. These result in increased expression of some of these immature-neuron-associated markers, which in turn could provide false positive or abnormally inflated results for “neurogenic activity” without involvement in actual NSCs or precursors [258].

## 6. Non-Canonical Neurogenic Sites

It should be noted that the two canonical NSC niches might not be the only source of neurogenic activity present in the brain. Potential alternative locations have also been described, such as the neocortex and the hypothalamus, but the relevance and magnitude of activity with respect to the main niches is not clear [259,260,261]. Similarly, a possible reservoir of undifferentiated progenitors has been found in the adult cerebellum, which respond to tissue degeneration [262] and may be able to differentiate and replenish the population of Purkinje cells after injury [263]. Other suggested sites of potential non-canonical-niche neurogenesis include amygdala [264,265], striatum [266], and substantia nigra [267]. The lack of information or certainty pertaining to some of these potential new sites can be attributed to current techniques that are not suitable for detecting the very limited amount of neurogenesis that takes place in basal conditions [268].

Many questions are yet to be answered, such as the origin of the progenitor cells that carry out these processes, and their possible relation or lineage connection to other (canonical) stem cell types. The significance of the processes may need questioning as well, since the resulting immature neurons might present impaired differentiation and survival and no clearly discernible role [269]. It is even called into question whether these constitute real adult neurogenic activity, as the evidence may only be the result of interference from dormant immature or “dematurated” mature neurons (explained in the previous section). While plasticity does seem to exist in these areas in one way or another and actual neurogenic activity cannot be ruled out as its source, it cannot be confirmed [253]. Therefore, the topic of neurogenesis outside of the canonical niches remains controversial.

## 7. Closing Remarks

We have progressed from the original dogma of a static nervous system beyond embryonic development, and a plethora of discoveries have been made on the dynamics of adult neurogenesis, NSCs, and their niches. Nevertheless, many questions remain unanswered.

Neurogenic activity on the canonical neural stem cell niches of adult mammals is widely accepted as a fact, and enough evidence supports this notion. Similarly, the basis of NSC as well as niche structure and dynamics seem to be understood to a reasonable extent. However, the details are not all clear. For example, whether all answers found in the study of animal models also translate to humans, including SVZ and OB neurogenesis (or possible lack thereof), or whether adult neurogenesis takes place in the human DG, as many studies suggest but others question. However, these niches are not exclusively composed by the NSCs and progenitors that reside and act inside of them, as there are plenty of other pieces in the puzzle which are only starting to be discovered. Elements, such as the vascular network, microglia, and even only the extracellular matrix of the niche itself, have relevant roles in neurogenesis regulation and maintenance.

The true role and relevance of NSCs in brain injury and pathological processes are also still under research, with certain intriguing results. For example, the effect of aging on NSC as well as niche function and structure, and its relationship with neurodegenerative disorders, which could eventually pave the way for new therapeutic approaches to alleviate the negative effects and risks of disease brought by the natural aging process.

To date, the spotlight has been placed on these canonical niches, and for good reason, as they are the expected main hotbeds of neurogenic activity, and the most likely ones to provide insight into NSC activity and dynamics (as well as the less difficult avenues to pursue that insight). Nevertheless, there are enough hints at other sources of neurogenesis and regeneration throughout the whole adult brain to not let the niches be the only focus, at least until a more definitive answer is found on the topic of whether adult neurogenesis really exists beyond the canonical niches. Any potential discoveries on this topic might be relevant in the study of brain dynamics in both health and pathology.

## Data Availability

Not applicable.

## References

[1] Altman J., Das G.D. (1965). Autoradiographic and histological evidence of postnatal hippocampal neurogenesis in rats. J. Comp. Neurol..

[2] Reynolds B.A., Weiss S. (1992). Generation of neurons and astrocytes from isolated cells of the adult mammalian central nervous system. Science.

[3] Eriksson P.S., Perfilieva E., Björk-Eriksson T., Alborn A.M., Nordborg C., Peterson D.A., Gage F.H. (1998). Neurogenesis in the adult human hippocampus. Nat. Med..

[4] Knoth R., Singec I., Ditter M., Pantazis G., Capetian P., Meyer R.P., Horvat V., Volk B., Kempermann G. (2010). Murine features of neurogenesis in the human hippocampus across the lifespan from 0 to 100 years. PLoS ONE.

[5] Boldrini M., Fulmore C.A., Tartt A.N., Simeon L.R., Pavlova I., Poposka V., Rosoklija G.B., Stankov A., Arango V., Dwork A.J. (2018). Human hippocampal neurogenesis persists throughout aging. Cell Stem Cell.

[6] Vicidomini C., Guo N., Sahay A. (2020). Communication, cross talk, and signal integration in the adult hippocampal neurogenic niche. Neuron.

[7] Gillotin S., Sahni V., Lepko T., Hanspal M.A., Swartz J.E., Alexopoulou Z., Marshall F.H. (2021). Targeting impaired adult hippocampal neurogenesis in ageing by leveraging intrinsic mechanisms regulating neural stem cell activity. Ageing Res. Rev..

[8] Moreno-Jiménez E.P., Terreros-Roncal J., Flor-García M., Rábano A., Llorens-Martín M. (2021). Evidences for adult hippocampal neurogenesis in humans. J. Neurosci..

[9] Spalding K.L., Bergmann O., Alkass K., Bernard S., Salehpour M., Huttner H.B., Boström E., Westerlund I., Vial C., Buchholz B.A. (2013). XDynamics of hippocampal neurogenesis in adult humans. Cell.

[10] Shors T.J., Miesegaes G., Beylin A., Zhao M., Rydel T., Gould E. (2001). Neurogenesis in the adult is involved in the formation of trace memories. Nature.

[11] Wojtowicz J.M., Askew M.L., Winocur G. (2008). The effects of running and of inhibiting adult neurogenesis on learning and memory in rats. Eur. J. Neurosci..

[12] Lagace D.C., Donovan M.H., Decarolis N.A., Farnbauch L.A., Malhotra S., Berton O., Nestler E.J., Krishnan V., Eisch A.J. (2010). Adult hippocampal neurogenesis is functionally important for stress-induced social avoidance. Proc. Natl. Acad. Sci. USA.

[13] Mak G.K., Weiss S. (2010). Paternal recognition of adult offspring mediated by newly generated CNS neurons. Nat. Neurosci..

[14] Feierstein C.E., Lazarini F., Wagner S., Gabellec M.M., de Chaumont F., Olivo-Marin J.C., Boussin F.D., Lledo P.M., Gheusi G. (2010). Disruption of adult neurogenesis in the olfactory bulb affects social interaction but not maternal behavior. Front. Behav. Neurosci..

[15] Sahay A., Scobie K.N., Hill A.S., O’Carroll C.M., Kheirbek M.A., Burghardt N.S., Fenton A.A., Dranovsky A., Hen R. (2011). Increasing adult hippocampal neurogenesis is sufficient to improve pattern separation. Nature.

[16] Snyder J.S., Soumier A., Brewer M., Pickel J., Cameron H.A. (2011). Adult hippocampal neurogenesis buffers stress responses and depressive behaviour. Nature.

[17] Kheirbek M.A., Tannenholz L., Hen R. (2012). NR2B-dependent plasticity of adult-born granule cells is necessary for context discrimination. J. Neurosci..

[18] Nakashiba T., Cushman J.D., Pelkey K.A., Renaudineau S., Buhl D.L., McHugh T.J., Barrera V.R., Chittajallu R., Iwamoto K.S., McBain C.J. (2012). Young dentate granule cells mediate pattern separation, whereas old granule cells facilitate pattern completion. Cell.

[19] Gómez-Gaviro M.V., Scott C.E., Sesay A.K., Matheu A., Booth S., Galichet C., Lovell-Badge R. (2012). Betacellulin promotes cell proliferation in the neural stem cell niche and stimulates neurogenesis. Proc. Natl. Acad. Sci. USA.

[20] Gómez-Gaviro M.V., Lovell-Badge R., Fernández-Avilés F., Lara-Pezzi E. (2012). The vascular stem cell niche. J. Cardiovasc. Transl. Res..

[21] Riquelme P.A., Drapeau E., Doetsch F. (2008). Brain micro-ecologies: Neural stem cell niches in the adult mammalian brain. Philos. Trans. R. Soc. B Biol. Sci..

[22] Wang C., Liu F., Liu Y.Y., Zhao C.H., You Y., Wang L., Zhang J., Wei B., Ma T., Zhang Q. (2011). Identification and characterization of neuroblasts in the subventricular zone and rostral migratory stream of the adult human brain. Cell Res..

[23] Ponti G.P., Obernier K., Alvarez-Buylla A. (2013). Lineage progression from stem cells to new neurons in the adult brain ventricularsubventricular zone. Cell Cycle.

[24] Kukekov V.G., Laywell E.D., Suslov O., Davies K., Scheffler B., Thomas L.B., O’Brien T.F., Kusakabe M., Steindler D.A. (1999). Multipotent stem/progenitor cells with similar properties arise from neurogenic regions of adult human brain. Exp. Neurol..

[25] Pencea V., Bingaman K.D., Freedman L.J., Luskin M.B. (2001). Neurogenesis in the subventricular zone and rostral migratory stream of the neonatal and adult primate forebrain. Exp. Neurol..

[26] Alvarez-Buylla A., García-Verdugo J.M. (2002). Neurogenesis in adult subventricular zone. J. Neurosci..

[27] Parent J.M. (2003). Injury-induced neurogenesis in the adult mammalian brain. Neuroscientist.

[28] Komitova M., Mattsson B., Johansson B.B., Eriksson P.S. (2005). Enriched environment increases neural stem/progenitor cell proliferation and neurogenesis in the subventricular zone of stroke-lesioned adult rats. Stroke.

[29] Liu H.K., Belz T., Bock D., Takacs A., Wu H., Lichter P., Chai M., Schütz G. (2008). The nuclear receptor tailless is required for neurogenesis in the adult subventricular zone. Genes Dev..

[30] López-Juárez A., Howard J., Ullom K., Howard L., Grande A., Pardo A., Waclaw R., Sun Y.Y., Yang D., Kuan C.Y. (2013). Gsx2 controls region-specific activation of neural stem cells and injury-induced neurogenesis in the adult subventricular zone. Genes Dev..

[31] Shen Q., Wang Y., Kokovay E., Lin G., Chuang S.M., Goderie S.K., Roysam B., Temple S. (2008). Adult SVZ stem cells lie in a vascular niche: A quantitative analysis of niche cell-cell interactions. Cell Stem Cell.

[32] Akter M., Kaneko N., Sawamoto K. (2021). Neurogenesis and neuronal migration in the postnatal ventricular-subventricular zone: Similarities and dissimilarities between rodents and primates. Neurosci. Res..

[33] Doetsch F., Scharff C. (2001). Challenges for brain repair: Insights from adult neurogenesis in birds and mammals. Brain. Behav. Evol..

[34] Mirzadeh Z., Merkle F.T., Soriano-Navarro M., Garcia-Verdugo J.M., Alvarez-Buylla A. (2008). Neural stem cells confer unique pinwheel architecture to the ventricular surface in neurogenic regions of the adult brain. Cell Stem Cell.

[35] Zhao X., Fisher E.S., Wang Y., Zuloaga K., Manley L., Temple S. (2022). 4D imaging analysis of the aging mouse neural stem cell niche reveals a dramatic loss of progenitor cell dynamism regulated by the RHO-ROCK pathway. Stem Cell Rep..

[36] Mizrak D., Bayin N.S., Yuan J., Liu Z., Suciu R.M., Niphakis M.J., Ngo N., Lum K.M., Cravatt B.F., Joyner A.L. (2020). Single-cell profiling and SCOPE-Seq reveal lineage dynamics of adult ventricular-subventricular zone neurogenesis and NOTUM as a key regulator. Cell Rep..

[37] Mizrak D., Levitin H.M., Delgado A.C., Crotet V., Yuan J., Chaker Z., Silva-Vargas V., Sims P.A., Doetsch F. (2019). Single-cell analysis of regional differences in adult V-SVZ neural stem cell lineages. Cell Rep..

[38] Cebrian-Silla A., Nascimento M.A., Redmond S.A., Mansky B., Wu D., Obernier K., Rodriguez R.R., Gonzalez-Granero S., Garcia-Verdugo J.M., Lim D.A. (2021). Single-cell analysis of the ventricular-subventricular zone reveals signatures of dorsal and ventral adult neurogenic lineages. Elife.

[39] Sawamoto K., Wichterle H., Gonzalez-Perez O., Cholfin J.A., Yamada M., Spassky N., Murcia N.S., Garcia-Verdugo J.M., Marin O., Rubenstein J.L.R. (2006). New neurons follow the flow of cerebrospinal fluid in the adult brain. Science.

[40] Bovetti S., Hsieh Y.C., Bovolin P., Perroteau I., Kazunori T., Puche A.C. (2007). Blood vessels form a scaffold for neuroblast migration in the adult olfactory bulb. J. Neurosci..

[41] Gengatharan A., Bammann R.R., Saghatelyan A. (2016). The role of astrocytes in the generation, migration, and integration of new neurons in the adult olfactory bulb. Front. Neurosci..

[42] Sanai N., Nguyen T., Ihrie R.A., Mirzadeh Z., Tsai H.H., Wong M., Gupta N., Berger M.S., Huang E., Garcia-Verdugo J.M. (2011). Corridors of migrating neurons in the human brain and their decline during infancy. Nature.

[43] Paredes M.F., James D., Gil-Perotin S., Kim H., Cotter J.A., Ng C., Sandoval K., Rowitch D.H., Xu D., McQuillen P.S. (2016). Extensive migration of young neurons into the infant human frontal lobe. Science.

[44] Rombaux P., Mouraux A., Bertrand B., Nicolas G., Duprez T., Hummel T. (2006). Olfactory function and olfactory bulb volume in patients with postinfectious olfactory loss. Laryngoscope.

[45] Rombaux P., Mouraux A., Bertrand B., Nicolas G., Duprez T., Hummel T. (2006). Retronasal and orthonasal olfactory function in relation to olfactory bulb volume in patients with posttraumatic loss of smell. Laryngoscope.

[46] Gudziol V., Buschhüter D., Abolmaali N., Gerber J., Rombaux P., Hummel T. (2009). Increasing olfactory bulb volume due to treatment of chronic rhinosinusitis-a longitudinal study. Brain.

[47] Pagano S.F., Impagnatiello F., Girelli M., Cova L., Grioni E., Onofri M., Cavallaro M., Etteri S., Vitello F., Giombini S. (2000). Isolation and characterization of neural stem cells from the adult human olfactory bulb. Stem Cells.

[48] Bergmann O., Liebl J., Bernard S., Alkass K., Yeung M.S.Y., Steier P., Kutschera W., Johnson L., Landén M., Druid H. (2012). The age of olfactory bulb neurons in humans. Neuron.

[49] Sanai H., Tramontin A.D., Quiñones-Hinojosa A., Barbaro N.M., Gupta H., Kunwar S., Lawton M.T., McDermott M.W., Parsa A.T., Verdugo J.M.G. (2004). Unique astrocyte ribbon in adult human brain contains neural stem cells but lacks chain migration. Nature.

[50] Curtis M.A., Kam M., Nannmark U., Anderson M.F., Axell M.Z., Wikkelso C., Holtås S., Van Roon-Mom W.M.C., Björk-Eriksson T., Nordborg C. (2007). Human neuroblasts migrate to the olfactory bulb via a lateral ventricular extension. Science.

[51] Burmeister H.P., Bitter T., Baltzer P.A.T., Dietzel M., Guntinas-Lichius O., Gudziol H., Kaiser W.A. (2011). Olfactory bulb ventricles as a frequent finding-a myth or reality? Evaluation using high resolution 3 Tesla magnetic resonance imaging. Neuroscience.

[52] Pozzati E., Martinoni M., Marucci G., Bacci A. (2014). Olfactory neuroblastoma and olfactory ventricle: A case report. Neuroradiol. J..

[53] Palmer T.D., Willhoite A.R., Gage F.H. (2000). Vascular niche for adult hippocampal neurogenesis. J. Comp. Neurol..

[54] Sun G.J., Zhou Y., Stadel R.P., Moss J., Yong J.H.A., Ito S., Kawasaki N.K., Phan A.T., Oh J.H., Modak N. (2015). Tangential migration of neuronal precursors of glutamatergic neurons in the adult mammalian brain. Proc. Natl. Acad. Sci. USA.

[55] Moss J., Gebara E., Bushong E.A., Sánchez-Pascual I., O’Laoi R., El M’Gharia I., Kocher-Braissant J., Ellisman M.H., Toni N. (2016). Fine processes of nestin-GFP-positive radial glia-like stem cells in the adult dentate gyrus ensheathe local synapses and vasculature. Proc. Natl. Acad. Sci. USA.

[56] Amrein I. (2015). Adult hippocampal neurogenesis in natural populations of mammals. Cold Spring Harb. Perspect. Biol..

[57] Nicola Z., Fabel K., Kempermann G. (2015). Development of the adult neurogenic niche in the hippocampus of mice. Front. Neuroanat..

[58] Winkelman M.A., Koppes A.N., Koppes R.A., Dai G. (2021). Bioengineering the neurovascular niche to study the interaction of neural stem cells and endothelial cells. APL Bioeng..

[59] Semënov M.V. (2019). Adult hippocampal neurogenesis is a developmental process involved in cognitive development. Front. Neurosci..

[60] Yassa M.A., Stark C.E.L. (2011). Pattern separation in the hippocampus. Trends Neurosci..

[61] Aimone J.B., Wiles J., Gage F.H. (2009). Computational influence of adult neurogenesis on memory encoding. Neuron.

[62] Appleby P.A., Wiskott L. (2009). Additive neurogenesis as a strategy for avoiding interference in a sparsely-coding dentate gyrus. Netw. Comput. Neural Syst..

[63] Appleby P.A., Kempermann G., Wiskott L. (2011). The role of additive neurogenesis and synaptic plasticity in a hippocampal memory model with grid-cell like input. PLoS Comput. Biol..

[64] Hollands C., Tobin M.K., Hsu M., Musaraca K., Yu T.S., Mishra R., Kernie S.G., Lazarov O. (2017). Depletion of adult neurogenesis exacerbates cognitive deficits in Alzheimer’s disease by compromising hippocampal inhibition. Mol. Neurodegener..

[65] Cope E.C., Waters R.C., Diethorn E.J., Pagliai K.A., Dias C.G., Tsuda M., Cameron H.A., Gould E. (2020). Adult-born neurons in the hippocampus are essential for social memory maintenance. eNeuro.

[66] Fuentealba L.C., Obernier K., Alvarez-Buylla A. (2012). Adult neural stem cells bridge their niche. Cell Stem Cell.

[67] Berg D.A., Su Y., Jimenez-Cyrus D., Patel A., Huang N., Morizet D., Lee S., Shah R., Ringeling F.R., Jain R. (2019). A common embryonic origin of stem cells drives developmental and adult neurogenesis. Cell.

[68] Bond A.M., Ming G., Song H. (2021). Ontogeny of adult neural stem cells in the mammalian brain. Curr. Top. Dev. Biol..

[69] Bonaguidi M.A., Wheeler M.A., Shapiro J.S., Stadel R.P., Sun G.J., Ming G.L., Song H. (2011). In vivo clonal analysis reveals self-renewing and multipotent adult neural stem cell characteristics. Cell.

[70] Seri B., García-Verdugo J.M., Collado-Morente L., McEwen B.S., Alvarez-Buylla A. (2004). Cell types, lineage, and architecture of the germinal zone in the adult dentate gyrus. J. Comp. Neurol..

[71] Filippov V., Kronenberg G., Pivneva T., Reuter K., Steiner B., Wang L.P., Yamaguchi M., Kettenmann H., Kempermann G. (2003). Subpopulation of nestin-expressing progenitor cells in the adult murine hippocampus shows electrophysiological and morphological characteristics of astrocytes. Mol. Cell. Neurosci..

[72] Licht T., Sasson E., Bell B., Grunewald M., Kumar S., Kreisel T., Ben-Zvi A., Keshet E. (2020). Hippocampal neural stem cells facilitate access from circulation via apical cytoplasmic processes. Elife.

[73] Dennis C.V., Suh L.S., Rodriguez M.L., Kril J.J., Sutherland G.T. (2016). Human adult neurogenesis across the ages: An immunohistochemical study. Neuropathol. Appl. Neurobiol..

[74] Sorrells S.F., Paredes M.F., Cebrian-Silla A., Sandoval K., Qi D., Kelley K.W., James D., Mayer S., Chang J., Auguste K.I. (2018). Human hippocampal neurogenesis drops sharply in children to undetectable levels in adults. Nature.

[75] Franjic D., Skarica M., Ma S., Arellano J.I., Tebbenkamp A.T.N., Choi J., Xu C., Li Q., Morozov Y.M., Andrijevic D. (2022). Transcriptomic taxonomy and neurogenic trajectories of adult human, macaque, and pig hippocampal and entorhinal cells. Neuron.

[76] Zhou Y., Su Y., Li S., Kennedy B.C., Zhang D.Y., Bond A.M., Sun Y., Jacob F., Lu L., Hu P. (2022). Molecular landscapes of human hippocampal immature neurons across lifespan. Nature.

[77] Flor-García M., Terreros-Roncal J., Moreno-Jiménez E.P., Ávila J., Rábano A., Llorens-Martín M. (2020). Unraveling human adult hippocampal neurogenesis. Nat. Protoc..

[78] Moreno-Jiménez E.P., Flor-García M., Terreros-Roncal J., Rábano A., Cafini F., Pallas-Bazarra N., Ávila J., Llorens-Martín M. (2019). Adult hippocampal neurogenesis is abundant in neurologically healthy subjects and drops sharply in patients with Alzheimer’s disease. Nat. Med..

[79] Paredes M.F., Sorrells S.F., Cebrian-Silla A., Sandoval K., Qi D., Kelley K.W., James D., Mayer S., Chang J., Auguste K.I. (2018). Does adult neurogenesis persist in the human hippocampus?. Cell Stem Cell.

[80] Lucassen P.J., Toni N., Kempermann G., Frisen J., Gage F.H., Swaab D.F. (2020). Limits to human neurogenesis—Really?. Mol. Psychiatry.

[81] Tartt A.N., Fulmore C.A., Liu Y., Rosoklija G.B., Dwork A.J., Arango V., Hen R., Mann J.J., Boldrini M. (2018). Considerations for assessing the extent of hippocampal neurogenesis in the adult and aging human brain. Cell Stem Cell.

[82] Lucassen P.J., Fitzsimons C.P., Salta E., Maletic-Savatic M. (2020). Adult neurogenesis, human after all (again): Classic, optimized, and future approaches. Behav. Brain Res..

[83] Song H., Stevens C.F., Gage F.H. (2002). Astroglia induce neurogenesis from adult neural stem cells. Nature.

[84] Armulik A., Genové G., Mäe M., Nisancioglu M.H., Wallgard E., Niaudet C., He L., Norlin J., Lindblom P., Strittmatter K. (2010). Pericytes regulate the blood-brain barrier. Nature.

[85] Tao Y., Ma L., Liao Z., Le Q., Yu J., Liu X., Li H., Chen Y., Zheng P., Yang Z. (2015). Astroglial β-arrestin1-mediated nuclear signaling regulates the expansion of neural precursor cells in adult hippocampus. Sci. Rep..

[86] Bell R.D., Winkler E.A., Sagare A.P., Singh I., LaRue B., Deane R., Zlokovic B.V. (2010). Pericytes control key neurovascular functions and neuronal phenotype in the adult brain and during brain aging. Neuron.

[87] Lacar B., Herman P., Platel J.C., Kubera C., Hyder F., Bordey A. (2012). Neural progenitor cells regulate capillary blood flow in the postnatal subventricular zone. J. Neurosci..

[88] Sofroniew M.V., Vinters H.V. (2010). Astrocytes: Biology and pathology. Acta Neuropathol..

[89] El Ali A., Rivest S. (2016). Microglia ontology and signaling. Front. Cell Dev. Biol..

[90] Ribeiro Xavier A.L., Kress B.T., Goldman S.A., De Lacerda Menezes J.R., Nedergaard M. (2015). A distinct population of microglia supports adult neurogenesis in the subventricular zone. J. Neurosci..

[91] Lopes K.D.P., Snijders G.J.L., Humphrey J., Allan A., Sneeboer M.A.M., Navarro E., Schilder B.M., Vialle R.A., Parks M., Missall R. (2022). Genetic analysis of the human microglial transcriptome across brain regions, aging and disease pathologies. Nat. Genet..

[92] Hohsfield L.A., Najafi A.R., Ghorbanian Y., Soni N., Crapser J.D., Figueroa Velez D.X., Jiang S., Royer S.E., Kim S.J., Henningfield C.M. (2021). Subventricular zone/white matter microglia reconstitute the empty adult microglial niche in a dynamic wave. Elife.

[93] Silva-Vargas V., Maldonado-Soto A.R., Mizrak D., Codega P., Doetsch F. (2016). Age-dependent niche signals from the choroid plexus regulate adult neural stem cells. Cell Stem Cell.

[94] Lee C., Hu J., Ralls S., Kitamura T., Loh Y.P., Yang Y., Mukouyama Y., Ahn S. (2012). The molecular profiles of neural stem cell niche in the adult subventricular zone. PLoS ONE.

[95] Gómez-Gaviro M.V., Desco M. (2018). The paracrine neural stem cell niche: New actors in the play. Curr. Stem Cell Rep..

[96] Spampinato S.F., Bortolotto V., Canonico P.L., Sortino M.A., Grilli M. (2019). Astrocyte-derived paracrine signals: Relevance for neurogenic niche regulation and blood-brain barrier integrity. Front. Pharmacol..

[97] Kadry H., Noorani B., Cucullo L. (2020). A blood–brain barrier overview on structure, function, impairment, and biomarkers of integrity. Fluids Barriers CNS.

[98] Ottone C., Krusche B., Whitby A., Clements M., Quadrato G., Pitulescu M.E., Adams R.H., Parrinello S. (2014). Direct cell-cell contact with the vascular niche maintains quiescent neural stem cells. Nat. Cell Biol..

[99] Kokovay E., Goderie S., Wang Y., Lotz S., Lin G., Sun Y., Roysam B., Shen Q., Temple S. (2010). Adult SVZ lineage cells home to and leave the vascular niche via differential responses to SDF1/CXCR4 signaling. Cell Stem Cell.

[100] Ramírez-Castillejo C., Sánchez-Sánchez F., Andreu-Agulló C., Ferrón S.R., Aroca-Aguilar J.D., Sánchez P., Mira H., Escribano J., Fariñas I. (2006). Pigment epithelium-derived factor is a niche signal for neural stem cell renewal. Nat. Neurosci..

[101] Obernier K., Alvarez-Buylla A. (2019). Neural stem cells: Origin, heterogeneity and regulation in the adult mammalian brain. Development.

[102] Gritti A., Parati E.A., Cova L., Frolichsthal P., Galli R., Wanke E., Faravelli L., Morassutti D.J., Roisen F., Nickel D.D. (1996). Multipotential stem cells from the adult mouse brain proliferate and self-renew in response to basic fibroblast growth factor. J. Neurosci..

[103] Zhang R., Zhang Z., Zhang C., Zhang L., Robin A., Wang Y., Lu M., Chopp M. (2004). Stroke transiently increases subventricular zone cell division from asymmetric to symmetric and increases neuronal differentiation in the adult rat. J. Neurosci..

[104] Zhang R.L., Chopp M., Roberts C., Liu X., Wei M., Nejad-Davarani S.P., Wang X., Zhang Z.G. (2014). Stroke increases neural stem cells and angiogenesis in the neurogenic niche of the adult mouse. PLoS ONE.

[105] Benner E.J., Luciano D., Jo R., Abdi K., Paez-Gonzalez P., Sheng H., Warner D.S., Liu C., Eroglu C., Kuo C.T. (2013). Protective astrogenesis from the SVZ niche after injury is controlled by Notch modulator Thbs4. Nature.

[106] Fuentealba L.C., Rompani S.B., Parraguez J.I., Obernier K., Romero R., Cepko C.L., Alvarez-Buylla A. (2015). Embryonic origin of postnatal neural stem cells. Cell.

[107] Codega P., Silva-Vargas V., Paul A., Maldonado-Soto A.R., DeLeo A.M., Pastrana E., Doetsch F. (2014). Prospective identification and purification of quiescent adult neural stem cells from their in vivo niche. Neuron.

[108] Boockvar J.A., Kapitonov D., Kapoor G., Schouten J., Counelis G.J., Bogler O., Snyder E.Y., McIntosh T.K., O’Rourke D.M. (2003). Constitutive EGFR signaling confers a motile phenotype to neural stem cells. Mol. Cell. Neurosci..

[109] Ayuso-Sacido A., Moliterno J.A., Kratovac S., Kapoor G.S., O’Rourke D.M., Holland E.C., Garci’a-Verdugo J.M., Roy N.S., Boockvar J.A. (2010). Activated EGFR signaling increases proliferation, survival, and migration and blocks neuronal differentiation in post-natal neural stem cells. J. Neuro-Oncol..

[110] Huttner W.B., Kosodo Y. (2005). Symmetric versus asymmetric cell division during neurogenesis in the developing vertebrate central nervous system. Curr. Opin. Cell Biol..

[111] Morrison S.J., Kimble J. (2006). Asymmetric and symmetric stem-cell divisions in development and cancer. Nature.

[112] Obernier K., Cebrian-Silla A., Thomson M., Parraguez J.I., Anderson R., Guinto C., Rodas Rodriguez J., Garcia-Verdugo J.M., Alvarez-Buylla A. (2018). Adult neurogenesis is sustained by symmetric self-renewal and differentiation. Cell Stem Cell.

[113] Pilz G.A., Bottes S., Betizeau M., Jörg D.J., Carta S., Simons B.D., Helmchen F., Jessberger S. (2018). Live imaging of neurogenesis in the adult mouse hippocampus. Science.

[114] Urbán N., Van Den Berg D.L.C., Forget A., Andersen J., Demmers J.A.A., Hunt C., Ayrault O., Guillemot F. (2016). Return to quiescence of mouse neural stem cells by degradation of a proactivation protein. Science.

[115] Pevny L., Placzek M. (2005). SOX genes and neural progenitor identity. Curr. Opin. Neurobiol..

[116] Wegner M., Stolt C.C. (2005). From stem cells to neurons and glia: A Soxist’s view of neural development. Trends Neurosci..

[117] Sarkar A., Hochedlinger K. (2013). The sox family of transcription factors: Versatile regulators of stem and progenitor cell fate. Cell Stem Cell.

[118] Kamachi Y., Kondoh H. (2013). Sox proteins: Regulators of cell fate specification and differentiation. Development.

[119] Schock E.N., LaBonne C. (2020). Sorting sox: Diverse roles for sox transcription factors during neural crest and craniofacial development. Front. Physiol..

[120] Lefebvre V. (2010). The SoxD transcription factors—Sox5, Sox6, and Sox13—Are key cell fate modulators. Int. J. Biochem. Cell Biol..

[121] Stolt C.C., Lommes P., Hillgärtner S., Wegner M. (2008). The transcription factor Sox5 modulates Sox10 function during melanocyte development. Nucleic Acids Res..

[122] Tanaka S., Suto A., Iwamoto T., Kashiwakuma D., Kagami S., Suzuki K., Takatori H., Tamachi T., Hirose K., Onodera A. (2014). Sox5 and C-Maf cooperatively induce Th17 cell differentiation via RORγt induction as downstream targets of Stat3. J. Exp. Med..

[123] Hagiwara N. (2011). Sox6, jack of all trades: A versatile regulatory protein in vertebrate development. Dev. Dyn..

[124] Wang Y., Bagheri-Fam S., Harley V.R. (2005). SOX13 is up-regulated in the developing mouse neuroepithelium and identifies a sub-population of differentiating neurons. Dev. Brain Res..

[125] Roose J., Korver W., Oving E., Wilson A., Wagenaar G., Markman M., Lamers W., Clevers H. (1998). High expression of the HMG box factor Sox-13 in arterial walls during embryonic development. Nucleic Acids Res..

[126] Melichar H.J., Narayan K., Der S.O., Hiraoka Y., Gardiol N., Jeannet G., Held W., Chambers C.A., Kang J. (2007). Regulation of Γδ versus Aβ T lymphocyte differentiation by the transcription factor SOX13. Science.

[127] Leone D.P., Srinivasan K., Chen B., Alcamo E., McConnell S.K. (2008). The determination of projection neuron identity in the developing cerebral cortex. Curr. Opin. Neurobiol..

[128] Azim E., Jabaudon D., Fame R.M., MacKlis J.D. (2009). SOX6 controls dorsal progenitor identity and interneuron diversity during neocortical development. Nat. Neurosci..

[129] Baroti T., Schillinger A., Wegner M., Stolt C.C. (2016). Sox13 functionally complements the related Sox5 and Sox6 as important developmental modulators in mouse spinal cord oligodendrocytes. J. Neurochem..

[130] Li L., Medina-Menéndez C., García-Corzo L., Córdoba-Beldad C.M., Quiroga A.C., Calleja Barca E., Zinchuk V., Muñoz-López S., Rodríguez-Martín P., Ciorraga M. (2022). SoxD genes are required for adult neural stem cell activation. Cell Rep..

[131] Mercurio S., Serra L., Nicolis S.K. (2019). More than just stem cells: Functional roles of the transcription factor Sox2 in differentiated glia and neurons. Int. J. Mol. Sci..

[132] Stolt C.C., Wegner M. (2010). SoxE function in vertebrate nervous system development. Int. J. Biochem. Cell Biol..

[133] Scott C.E., Wynn S.L., Sesay A., Cruz C., Cheung M., Gaviro M.V.G., Booth S., Gao B., Cheah K.S.E., Lovell-Badge R. (2010). SOX9 induces and maintains neural stem cells. Nat. Neurosci..

[134] Cheng L.C., Pastrana E., Tavazoie M., Doetsch F. (2009). MiR-124 regulates adult neurogenesis in the subventricular zone stem cell niche. Nat. Neurosci..

[135] Mokabber H., Najafzadeh N., Mohammadzadeh Vardin M. (2019). MiR-124 promotes neural differentiation in mouse bulge stem cells by repressing Ptbp1 and Sox9. J. Cell. Physiol..

[136] Potzner M.R., Tsarovina K., Binder E., Penzo-Méndez A., Lefebvre V., Rohrer H., Wegner M., Sock E. (2010). Sequential requirement of Sox4 and Sox11 during development of the sympathetic nervous system. Development.

[137] Thein D.C., Thalhammer J.M., Hartwig A.C., Bryan Crenshaw E., Lefebvre V., Wegner M., Sock E. (2010). The closely related transcription factors Sox4 and Sox11 function as survival factors during spinal cord development. J. Neurochem..

[138] Bergsland M., Ramsköld D., Zaouter C., Klum S., Sandberg R., Muhr J. (2011). Sequentially acting sox transcription factors in neural lineage development. Genes Dev..

[139] Mu L., Berti L., Masserdotti G., Covic M., Michaelidis T.M., Doberauer K., Merz K., Rehfeld F., Haslinger A., Wegner M. (2012). SoxC transcription factors are required for neuronal differentiation in adult hippocampal neurogenesis. J. Neurosci..

[140] Rao M.S., Shetty A.K. (2004). Efficacy of doublecortin as a marker to analyse the absolute number and dendritic growth of newly generated neurons in the adult dentate gyrus. Eur. J. Neurosci..

[141] Ayanlaja A.A., Xiong Y., Gao Y., Ji G., Tang C., Abdullah Z., Gao D. (2017). Distinct features of doublecortin as a marker of neuronal migration and its implications in cancer cell mobility. Front. Mol. Neurosci..

[142] Couillard-Despres S., Winner B., Schaubeck S., Aigner R., Vroemen M., Weidner N., Bogdahn U., Winkler J., Kuhn H.G., Aigner L. (2005). Doublecortin expression levels in adult brain reflect neurogenesis. Eur. J. Neurosci..

[143] Brown J.P., Couillard-Després S., Cooper-Kuhn C.M., Winkler J., Aigner L., Kuhn H.G. (2003). Transient expression of doublecortin during adult neurogenesis. J. Comp. Neurol..

[144] Bernal A., Arranz L. (2018). Nestin-expressing progenitor cells: Function, identity and therapeutic implications. Cell. Mol. Life Sci..

[145] Mignone J.L., Kukekov V., Chiang A.S., Steindler D., Enikolopov G. (2004). Neural stem and progenitor cells in nestin-GFP transgenic mice. J. Comp. Neurol..

[146] Bott C.J., Johnson C.G., Yap C.C., Dwyer N.D., Litwa K.A., Winckler B. (2019). Nestin in immature embryonic neurons affects axon growth cone morphology and Semaphorin3a sensitivity. Mol. Biol. Cell.

[147] Sun B., Chang E., Gerhartl A., Szele F.G. (2018). Polycomb protein eed is required for neurogenesis and cortical injury activation in the subventricular zone. Cereb. Cortex.

[148] Yu Y., Chen Y., Kim B., Wang H., Zhao C., He X., Liu L., Liu W., Wu L.M.N., Mao M. (2013). Olig2 targets chromatin remodelers to enhancers to initiate oligodendrocyte differentiation. Cell.

[149] Ligon K.L., Huillard E., Mehta S., Kesari S., Liu H., Alberta J.A., Bachoo R.M., Kane M., Louis D.N., DePinho R.A. (2007). Olig2-regulated lineage-restricted pathway controls replication competence in neural stem cells and malignant glioma. Neuron.

[150] Setoguchi T., Kondo T. (2004). Nuclear export of OLIG2 in neural stem cells is essential for ciliary neurotrophic factor-induced astrocyte differentiation. J. Cell Biol..

[151] Semerci F., Tin-Shing Choi W., Bajic A., Thakkar A., Encinas J.M., Depreux F., Segil N., Groves A.K., Maletic-Savatic M. (2017). Lunatic fringe-mediated notch signaling regulates adult hippocampal neural stem cell maintenance. Elife.

[152] Anam M.B., Ahmad S.A.I., Kudo M., Istiaq A., Felemban A.A.M., Ito N., Ohta K. (2020). Akhirin regulates the proliferation and differentiation of neural stem cells/progenitor cells at neurogenic niches in mouse brain. Dev. Growth Differ..

[153] Kudo M., Ohta K. (2021). Regulation of the brain neural niche by soluble molecule akhirin. J. Dev. Biol..

[154] Manganas L.N., Durá I., Osenberg S., Semerci F., Tosun M., Mishra R., Parkitny L., Encinas J.M., Maletic-Savatic M. (2021). BASP1 labels neural stem cells in the neurogenic niches of mammalian brain. Sci. Rep..

[155] Bátiz L.F., Castro M.A., Burgos P.V., Velásquez Z.D., Munoz R.I., Lafourcade C.A., Troncoso-Escudero P., Wyneken U. (2016). Exosomes as novel regulators of adult neurogenic niches. Front. Cell. Neurosci..

[156] Jakubec M., Maple-Grødem J., Akbari S., Nesse S., Halskau Ø., Mork-Jansson A.E. (2020). Plasma-derived exosome-like vesicles are enriched in lyso-phospholipids and pass the blood-brain barrier. PLoS ONE.

[157] Selmaj I., Mycko M.P., Raine C.S., Selmaj K.W. (2017). The role of exosomes in CNS inflammation and their involvement in multiple sclerosis. J. Neuroimmunol..

[158] Zappulli V., Pagh Friis K., Fitzpatrick Z., Maguire C.A., Breakefield X.O. (2016). Extracellular vesicles and intercellular communication within the nervous system. J. Clin. Investig..

[159] Frühbeis C., Fröhlich D., Kuo W.P., Amphornrat J., Thilemann S., Saab A.S., Kirchhoff F., Möbius W., Goebbels S., Nave K.A. (2013). Neurotransmitter-triggered transfer of exosomes mediates oligodendrocyte-neuron communication. PLoS Biol..

[160] Zhang Y.Z., Liu F., Song C.G., Cao X.L., Zhang Y.F., Wu H.N., Guo C.J., Li Y.Q., Zheng Q.J., Zheng M.H. (2018). Exosomes derived from human umbilical vein endothelial cells promote neural stem cell expansion while maintain their stemness in culture. Biochem. Biophys. Res. Commun..

[161] Zhang Y., Chopp M., Meng Y., Katakowski M., Xin H., Mahmood A., Xiong Y. (2015). Effect of exosomes derived from multipluripotent mesenchymal stromal cells on functional recovery and neurovascular plasticity in rats after traumatic brain injury. J. Neurosurg..

[162] Cossetti C., Iraci N., Mercer T.R., Leonardi T., Alpi E., Drago D., Alfaro-Cervello C., Saini H.K., Davis M.P., Schaeffer J. (2014). Extracellular vesicles from neural stem cells transfer IFN-γ via Ifngr1 to activate Stat1 signaling in target cells. Mol. Cell.

[163] Webb R.L., Kaiser E.E., Jurgielewicz B.J., Spellicy S., Scoville S.L., Thompson T.A., Swetenburg R.L., Hess D.C., West F.D., Stice S.L. (2018). Human neural stem cell extracellular vesicles improve recovery in a porcine model of ischemic stroke. Stroke.

[164] Sun M.K., Passaro A.P., Latchoumane C.F., Spellicy S.E., Bowler M., Goeden M., Martin W.J., Holmes P.V., Stice S.L., Karumbaiah L. (2020). Extracellular vesicles mediate neuroprotection and functional recovery after traumatic brain injury. J. Neurotrauma.

[165] Adlakha Y.K., Saini N. (2014). Brain MicroRNAs and insights into biological functions and therapeutic potential of brain enriched MiRNA-128. Mol. Cancer.

[166] Bartel D.P. (2004). MicroRNAs: Genomics, biogenesis, mechanism, and function. Cell.

[167] Lagos-Quintana M., Rauhut R., Yalcin A., Meyer J., Lendeckel W., Tuschl T. (2002). Identification of tissue-specific MicroRNAs from mouse. Curr. Biol..

[168] Zhang Y., Kim M.S., Jia B., Yan J., Zuniga-Hertz J.P., Han C., Cai D. (2017). Hypothalamic stem cells control ageing speed partly through exosomal MiRNAs. Nature.

[169] Yuan P., Ding L., Chen H., Wang Y., Li C., Zhao S., Yang X., Ma Y., Zhu J., Qi X. (2021). Neural stem cell-derived exosomes regulate neural stem cell differentiation through MiR-9-Hes1 axis. Front. Cell Dev. Biol..

[170] Hou B.R., Jiang C., Wang Z.N., Ren H.J. (2020). Exosome-mediated crosstalk between microglia and neural stem cells in the repair of brain injury. Neural Regen. Res..

[171] Morton M.C., Neckles V.N., Seluzicki C.M., Holmberg J.C., Feliciano D.M. (2018). Neonatal subventricular zone neural stem cells release extracellular vesicles that act as a microglial morphogen. Cell Rep..

[172] Lepko T., Pusch M., Müller T., Schulte D., Ehses J., Kiebler M., Hasler J., Huttner H.B., Vandenbroucke R.E., Vandendriessche C. (2019). Choroid plexus-derived MiR-204 regulates the number of quiescent neural stem cells in the adult brain. EMBO J..

[173] Frisén J. (2016). Neurogenesis and gliogenesis in nervous system plasticity and repair. Annu. Rev. Cell Dev. Biol..

[174] Barker R.A., Götz M., Parmar M. (2018). New approaches for brain repair—From rescue to reprogramming. Nature.

[175] Kerever A., Schnack J., Vellinga D., Ichikawa N., Moon C., Arikawa-Hirasawa E., Efird J.T., Mercier F. (2007). Novel extracellular matrix structures in the neural stem cell niche capture the neurogenic factor fibroblast growth factor 2 from the extracellular milieu. Stem Cells.

[176] Mercier F. (2016). Fractones: Extracellular matrix niche controlling stem cell fate and growth factor activity in the brain in health and disease. Cell. Mol. Life Sci..

[177] Pathak M.M., Nourse J.L., Tran T., Hwe J., Arulmoli J., Le D.T.T., Bernardis E., Flanagan L.A., Tombola F. (2014). Stretch-activated ion channel Piezo1 directs lineage choice in human neural stem cells. Proc. Natl. Acad. Sci. USA.

[178] Kjell J., Fischer-Sternjak J., Thompson A.J., Friess C., Sticco M.J., Salinas F., Cox J., Martinelli D.C., Ninkovic J., Franze K. (2020). Defining the adult neural stem cell niche proteome identifies key regulators of adult neurogenesis. Cell Stem Cell.

[179] Yayon A., Klagsbrun M., Esko J.D., Leder P., Ornitz D.M. (1991). Cell surface, heparin-like molecules are required for binding of basic fibroblast growth factor to its high affinity receptor. Cell.

[180] Douet V., Kerever A., Arikawa-Hirasawa E., Mercier F. (2013). Fractone-heparan sulphates mediate FGF-2 stimulation of cell proliferation in the adult subventricular zone. Cell Prolif..

[181] Douet V., Arikawa-Hirasawa E., Mercier F. (2012). Fractone-heparan sulfates mediate BMP-7 inhibition of cell proliferation in the adult subventricular zone. Neurosci. Lett..

[182] Mercier F., Douet V. (2014). Bone morphogenetic protein-4 inhibits adult neurogenesis and is regulated by fractone-associated heparan sulfates in the subventricular zone. J. Chem. Neuroanat..

[183] Nascimento M.A., Sorokin L., Coelho-Sampaio T. (2018). Fractone bulbs derive from ependymal cells and their laminin composition influence the stem cell niche in the subventricular zone. J. Neurosci..

[184] Brooker S.M., Bond A.M., Peng C.Y., Kessler J.A. (2016). Β1-integrin restricts astrocytic differentiation of adult hippocampal neural stem cells. Glia.

[185] Sakka L., Coll G., Chazal J. (2011). Anatomy and physiology of cerebrospinal fluid. Eur. Ann. Otorhinolaryngol. Head Neck Dis..

[186] Lun M.P., Monuki E.S., Lehtinen M.K. (2015). Development and functions of the choroid plexus-cerebrospinal fluid system. Nat. Rev. Neurosci..

[187] Fame R.M., Lehtinen M.K. (2020). Emergence and developmental roles of the cerebrospinal fluid system. Dev. Cell.

[188] Guerra M.M., González C., Caprile T., Jara M., Vío K., Muñoz R.I., Rodríguez S., Rodríguez E.M. (2015). Understanding how the subcommissural organ and other periventricular secretory structures contribute via the cerebrospinal fluid to neurogenesis. Front. Cell. Neurosci..

[189] Gato A., Desmond M.E. (2009). Why the embryo still matters: CSF and the neuroepithelium as interdependent regulators of embryonic brain growth, morphogenesis and histiogenesis. Dev. Biol..

[190] Alonso M.I., Martín C., Carnicero E., Bueno D., Gato A. (2011). Cerebrospinal fluid control of neurogenesis induced by retinoic acid during early brain development. Dev. Dyn..

[191] Alonso M.I., Lamus F., Carnicero E., Moro J.A., de la Mano A., Fernández J.M.F., Desmond M.E., Gato A. (2017). Embryonic cerebrospinal fluid increases neurogenic activity in the brain ventricular-subventricular zone of adult mice. Front. Neuroanat..

[192] Buddensiek J., Dressel A., Kowalski M., Runge U., Schroeder H., Hermann A., Kirsch M., Storch A., Sabolek M. (2010). Cerebrospinal fluid promotes survival and astroglial differentiation of adult human neural progenitor cells but inhibits proliferation and neuronal differentiation. BMC Neurosci..

[193] de Sonnaville S.F.A.M., van Strien M.E., Middeldorp J., Sluijs J.A., van den Berge S.A., Moeton M., Donega V., van Berkel A., Deering T., De Filippis L. (2020). The adult human subventricular zone: Partial ependymal coverage and proliferative capacity of cerebrospinal fluid. Brain Commun..

[194] Lehtinen M.K., Zappaterra M.W., Chen X., Yang Y.J., Hill A.D., Lun M., Maynard T., Gonzalez D., Kim S., Ye P. (2011). The cerebrospinal fluid provides a proliferative niche for neural progenitor cells. Neuron.

[195] Petrik D., Myoga M.H., Grade S., Gerkau N.J., Pusch M., Rose C.R., Grothe B., Götz M. (2018). Epithelial sodium channel regulates adult neural stem cell proliferation in a flow-dependent manner. Cell Stem Cell.

[196] Kaneko N., Sawamoto K. (2018). Go with the flow: Cerebrospinal fluid flow regulates neural stem cell proliferation. Cell Stem Cell.

[197] Lazarov O., Marr R.A. (2010). Neurogenesis and Alzheimer’s disease: At the crossroads. Exp. Neurol..

[198] Terreros-Roncal J., Moreno-Jiménez E.P., Flor-García M., Rodríguez-Moreno C.B., Trinchero M.F., Cafini F., Rábano A., Llorens-Martín M. (2021). Impact of neurodegenerative diseases on human adult hippocampal neurogenesis. Science.

[199] Winner B., Winkler J. (2015). Adult neurogenesis in neurodegenerative diseases. Cold Spring Harb. Perspect. Biol..

[200] Cho K.O., Lybrand Z.R., Ito N., Brulet R., Tafacory F., Zhang L., Good L., Ure K., Kernie S.G., Birnbaum S.G. (2015). Aberrant hippocampal neurogenesis contributes to epilepsy and associated cognitive decline. Nat. Commun..

[201] Horgusluoglu E., Nudelman K., Nho K., Saykin A.J. (2017). Adult neurogenesis and neurodegenerative diseases: A systems biology perspective. Am. J. Med. Genet. Part B Neuropsychiatr. Genet..

[202] Luo J., Daniels S.B., Lennington J.B., Notti R.Q., Conover J.C. (2006). The aging neurogenic subventricular zone. Aging Cell.

[203] Kuhn H.G., Dickinson-Anson H., Gage F.H. (1996). Neurogenesis in the dentate gyrus of the adult rat: Age-related decrease of neuronal progenitor proliferation. J. Neurosci..

[204] Enwere E., Shingo T., Gregg C., Fujikawa H., Ohta S., Weiss S. (2004). Aging results in reduced epidermal growth factor receptor signaling, diminished olfactory neurogenesis, and deficits in fine olfactory discrimination. J. Neurosci..

[205] Apple D.M., Solano-Fonseca R., Kokovay E. (2017). Neurogenesis in the aging brain. Biochem. Pharmacol..

[206] Villeda S.A., Luo J., Mosher K.I., Zou B., Britschgi M., Bieri G., Stan T.M., Fainberg N., Ding Z., Eggel A. (2011). The ageing systemic milieu negatively regulates neurogenesis and cognitive function. Nature.

[207] Smith L.K., He Y., Park J.S., Bieri G., Snethlage C.E., Lin K., Gontier G., Wabl R., Plambeck K.E., Udeochu J. (2015). Β2-microglobulin is a systemic pro-aging factor that impairs cognitive function and neurogenesis. Nat. Med..

[208] Katsimpardi L., Litterman N.K., Schein P.A., Miller C.M., Loffredo F.S., Wojtkiewicz G.R., Chen J.W., Lee R.T., Wagers A.J., Rubin L.L. (2014). Vascular and neurogenic rejuvenation of the aging mouse brain by young systemic factors. Science.

[209] Ozek C., Krolewski R.C., Buchanan S.M., Rubin L.L. (2018). Growth differentiation factor 11 treatment leads to neuronal and vascular improvements in the hippocampus of aged mice. Sci. Rep..

[210] Yousef H., Czupalla C.J., Lee D., Chen M.B., Burke A.N., Zera K.A., Zandstra J., Berber E., Lehallier B., Mathur V. (2019). Aged blood impairs hippocampal neural precursor activity and activates microglia via brain endothelial cell VCAM1. Nat. Med..

[211] Zhao X., Wang Y., Wait E., Mankowski W., Bjornsson C.S., Cohen A.R., Zuloaga K.L., Temple S. (2021). 3D Image analysis of the complete ventricular-subventricular zone stem cell niche reveals significant vasculature changes and progenitor deficits in males versus females with aging. Stem Cell Rep..

[212] Wang L., Zhao F., Wang D., Hu S., Liu J., Zhou Z., Lu J., Qi P., Song S. (2016). Pressure drop in tortuosity/kinking of the internal carotid artery: Simulation and clinical investigation. Biomed Res. Int..

[213] Culver J.C., Vadakkan T.J., Dickinson M.E. (2013). A specialized microvascular domain in the mouse neural stem cell niche. PLoS ONE.

[214] Sierra A., Encinas J.M., Deudero J.J.P., Chancey J.H., Enikolopov G., Overstreet-Wadiche L.S., Tsirka S.E., Maletic-Savatic M. (2010). Microglia shape adult hippocampal neurogenesis through apoptosis-coupled phagocytosis. Cell Stem Cell.

[215] Lin M.T., Beal M.F. (2006). Mitochondrial dysfunction and oxidative stress in neurodegenerative diseases. Nature.

[216] Chan Y.L.E., Bai Y.M., Hsu J.W., Huang K.L., Su T.P., Li C.T., Lin W.C., Pan T.L., Chen T.J., Tsai S.J. (2017). Post-traumatic stress disorder and risk of parkinson disease: A nationwide longitudinal study. Am. J. Geriatr. Psychiatry.

[217] Johansson L., Kern S., Zetterberg H., Blennow K., Börjesson-Hansson A., Rosengren L., Guo X., Skoog I. (2018). Midlife stress in relation to late-life cerebrospinal fluid biomarkers of Alzheimer’s disease: A 25-year follow-up study. Dement. Geriatr. Cogn. Disord..

[218] Egeland M., Zunszain P.A., Pariante C.M. (2015). Molecular mechanisms in the regulation of adult neurogenesis during stress. Nat. Rev. Neurosci..

[219] Jung S., Choe S., Woo H., Jeong H., An H.K., Moon H., Ryu H.Y., Yeo B.K., Lee Y.W., Choi H. (2020). Autophagic death of neural stem cells mediates chronic stress-induced decline of adult hippocampal neurogenesis and cognitive deficits. Autophagy.

[220] Rawat R., Tunc-Ozcan E., McGuire T.L., Peng C.-Y., Kessler J.A. (2022). Ketamine activates adult-born immature granule neurons to rapidly alleviate depression-like behaviors in mice. Nat. Commun..

[221] Kooi Ong L., Rohan Walker F., Nilsson M. (2017). Is stroke a neurodegenerative condition? A critical review of secondary neurodegeneration and amyloid-beta accumulation after stroke. AIMS Med. Sci..

[222] Tuazon J.P., Castelli V., Lee J.Y., Desideri G.B., Stuppia L., Cimini A.M., Borlongan C.V. (2019). Neural stem cells. Adv. Exp. Med. Biol..

[223] Sakata H., Niizuma K., Yoshioka H., Kim G.S., Jung J.E., Katsu M., Narasimhan P., Maier C.M., Nishiyama Y., Chan P.H. (2012). Minocycline-preconditioned neural stem cells enhance neuroprotection after ischemic stroke in rats. J. Neurosci..

[224] Kahroba H., Ramezani B., Maadi H., Sadeghi M.R., Jaberie H., Ramezani F. (2021). The role of Nrf2 in neural stem/progenitors cells: From maintaining stemness and self-renewal to promoting differentiation capability and facilitating therapeutic application in neurodegenerative disease. Ageing Res. Rev..

[225] Hamilton L.K., Dufresne M., Joppé S.E., Petryszyn S., Aumont A., Calon F., Barnabé-Heider F., Furtos A., Parent M., Chaurand P. (2015). Aberrant lipid metabolism in the forebrain niche suppresses adult neural stem cell proliferation in an animal model of Alzheimer’s disease. Cell Stem Cell.

[226] Montagne A., Barnes S.R., Sweeney M.D., Halliday M.R., Sagare A.P., Zhao Z., Toga A.W., Jacobs R.E., Liu C.Y., Amezcua L. (2015). Blood-brain barrier breakdown in the aging human hippocampus. Neuron.

[227] Pous L., Deshpande S.S., Nath S., Mezey S., Malik S.C., Schildge S., Bohrer C., Topp K., Pfeifer D., Fernández-Klett F. (2020). Fibrinogen induces neural stem cell differentiation into astrocytes in the subventricular zone via BMP signaling. Nat. Commun..

[228] Hayes M.T. (2019). Parkinson’s disease and parkinsonism. Am. J. Med..

[229] L’Episcopo F., Tirolo C., Peruzzotti-Jametti L., Serapide M.F., Testa N., Caniglia S., Balzarotti B., Pluchino S., Marchetti B. (2018). Neural stem cell grafts promote astroglia-driven neurorestoration in the aged parkinsonian brain via Wnt/β-Catenin signaling. Stem Cells.

[230] Zhang S.C., Ge B., Duncan I.D. (1999). Adult brain retains the potential to generate oligodendroglial progenitors with extensive myelination capacity. Proc. Natl. Acad. Sci. USA.

[231] Billon N., Jolicoeur C., Ying Q.L., Smith A., Raff M. (2002). Normal timing of oligodendrocyte development from genetically engineered, lineage-selectable mouse ES cells. J. Cell Sci..

[232] Chou C.H., Fan H.C., Hueng D.Y. (2015). Potential of neural stem cell-based therapy for Parkinson’s disease. Parkinson′s Dis..

[233] Davis M.E. (2016). Glioblastoma: Overview of disease and treatment. Clin. J. Oncol. Nurs..

[234] Tan A.C., Ashley D.M., López G.Y., Malinzak M., Friedman H.S., Khasraw M. (2020). Management of glioblastoma: State of the art and future directions. CA Cancer J. Clin..

[235] Jafri N.F., Clarke J.L., Weinberg V., Barani I.J., Cha S. (2013). Relationship of glioblastoma multiforme to the subventricular zone is associated with survival. Neuro. Oncol..

[236] Adeberg S., Bostel T., König L., Welzel T., Debus J., Combs S.E. (2014). A comparison of long-term survivors and short-term survivors with glioblastoma, subventricular zone involvement: A predictive factor for survival?. Radiat. Oncol..

[237] Khalifa J., Tensaouti F., Lusque A., Plas B., Lotterie J.A., Benouaich-Amiel A., Uro-Coste E., Lubrano V., Cohen-Jonathan Moyal E. (2017). Subventricular zones: New key targets for glioblastoma treatment. Radiat. Oncol..

[238] Berendsen S., Van Bodegraven E., Seute T., Spliet W.G.M., Geurts M., Hendrikse J., Schoysman L., Huiszoon W.B., Varkila M., Rouss S. (2019). Adverse prognosis of glioblastoma contacting the subventricular zone: Biological correlates. PLoS ONE.

[239] Smith A.W., Mehta M.P., Wernicke A.G. (2016). Neural stem cells, the subventricular zone and radiotherapy: Implications for treating glioblastoma. J. Neuro-Oncol..

[240] Nourallah B., Digpal R., Jena R., Watts C. (2017). Irradiating the subventricular zone in glioblastoma patients: Is there a case for a clinical trial?. Clin. Oncol..

[241] Liebelt B.D., Shingu T., Zhou X., Ren J., Shin S.A., Hu J. (2016). Glioma stem cells: Signaling, microenvironment, and therapy. Stem Cells Int..

[242] Gimple R.C., Bhargava S., Dixit D., Rich J.N. (2019). Glioblastoma stem cells: Lessons from the tumor hierarchy in a lethal cancer. Genes Dev..

[243] Lee J.H., Lee J.E., Kahng J.Y., Kim S.H., Park J.S., Yoon S.J., Um J.Y., Kim W.K., Lee J.K., Park J. (2018). Human glioblastoma arises from subventricular zone cells with low-level driver mutations. Nature.

[244] Matarredona E.R., Pastor A.M. (2019). Neural stem cells of the subventricular zone as the origin of human glioblastoma stem cells. Therapeutic implications. Front. Oncol..

[245] Bonfanti L., Peretto P. (2011). Adult neurogenesis in mammals—A theme with many variations. Eur. J. Neurosci..

[246] Lipp H.P., Bonfanti L. (2016). Adult neurogenesis in mammals: Variations and confusions. Brain Behav. Evol..

[247] Paredes M.F., Sorrells S.F., Garcia-Verdugo J.M., Alvarez-Buylla A. (2016). Brain size and limits to adult neurogenesis. J. Comp. Neurol..

[248] Rubio A., Belles M., Belenguer G., Vidueira S., Fariñas I., Nacher J. (2016). Characterization and isolation of immature neurons of the adult mouse piriform cortex. Dev. Neurobiol..

[249] Rotheneichner P., Belles M., Benedetti B., König R., Dannehl D., Kreutzer C., Zaunmair P., Engelhardt M., Aigner L., Nacher J. (2018). Cellular plasticity in the adult murine piriform cortex: Continuous maturation of dormant precursors into excitatory neurons. Cereb. Cortex.

[250] Piumatti M., Palazzo O., La Rosa C., Crociara P., Parolisi R., Luzzati F., Lévy F., Bonfanti L. (2018). Non-newly generated, “immature” neurons in the sheep brain are not restricted to cerebral cortex. J. Neurosci..

[251] Sorrells S.F., Paredes M.F., Velmeshev D., Herranz-Pérez V., Sandoval K., Mayer S., Chang E.F., Insausti R., Kriegstein A.R., Rubenstein J.L. (2019). Immature excitatory neurons develop during adolescence in the human amygdala. Nat. Commun..

[252] Benedetti B., Dannehl D., König R., Coviello S., Kreutzer C., Zaunmair P., Jakubecova D., Weiger T.M., Aigner L., Nacher J. (2020). Functional integration of neuronal precursors in the adult murine piriform cortex. Cereb. Cortex.

[253] Feliciano D.M., Bordey A., Bonfanti L. (2015). Noncanonical sites of adult neurogenesis in the mammalian brain. Cold Spring Harb. Perspect. Biol..

[254] Verdaguer E., García-Jordà E., Canudas A.M., Domínguez E., Jiménez A., Pubill D., Escubedo E., Pallàs J.C.M., Camins A. (2002). Kainic acid-induced apoptosis in cerebellar granule neurons: An attempt at cell cycle re-entry. Neuroreport.

[255] Negis Y., Karabay A. (2016). Expression of cell cycle proteins in cortical neurons—Correlation with glutamate-induced neurotoxicity. BioFactors.

[256] Kobayashi K., Ikeda Y., Sakai A., Yamasaki N., Haneda E., Miyakawa T., Suzuki H. (2010). Reversal of hippocampal neuronal maturation by serotonergic antidepressants. Proc. Natl. Acad. Sci. USA.

[257] Hagihara H., Ohira K., Miyakawa T. (2019). Transcriptomic evidence for immaturity induced by antidepressant fluoxetine in the hippocampus and prefrontal cortex. Neuropsychopharmacol. Rep..

[258] Hagihara H., Murano T., Ohira K., Miwa M., Nakamura K., Miyakawa T. (2019). Expression of progenitor cell/immature neuron markers does not present definitive evidence for adult neurogenesis. Mol. Brain.

[259] Cameron H.A., Dayer A.G. (2008). New interneurons in the adult neocortex: Small, sparse, but significant?. Biol. Psychiatry.

[260] Kokoeva M.V., Yin H., Flier J.S. (2005). Neurogenesis in the hypothalamus of adult mice: Potential role in energy balance. Science.

[261] Rojczyk-Gołębiewska E., Pałasz A., Wiaderkiewicz R. (2014). Hypothalamic subependymal niche: A novel site of the adult neurogenesis. Cell. Mol. Neurobiol..

[262] Salih S., Nizamudeen Z.A., De Melo N., Chakrabarti L., Sottile V. (2022). Sox-positive cell population in the adult cerebellum increases upon tissue degeneration. Exp. Neurol..

[263] Ahlfeld J., Filser S., Schmidt F., Wefers A.K., Merk D.J., Glaß R., Herms J., Schüller U. (2017). Neurogenesis from Sox2 expressing cells in the adult cerebellar cortex. Sci. Rep..

[264] Bernier P.J., Bédard A., Vinet J., Lévesque M., Parent A. (2002). Newly generated neurons in the amygdala and adjoining cortex of adult primates. Proc. Natl. Acad. Sci. USA.

[265] Roeder S.S., Burkardt P., Rost F., Rode J., Brusch L., Coras R., Englund E., Håkansson K., Possnert G., Salehpour M. (2022). Evidence for postnatal neurogenesis in the human amygdala. Commun. Biol..

[266] Bédard A., Gravel C., Parent A. (2006). Chemical characterization of newly generated neurons in the striatum of adult primates. Exp. Brain Res..

[267] Zhao M., Janson Lang A.M. (2009). Bromodeoxyuridine infused into the cerebral ventricle of adult mice labels nigral neurons under physiological conditions—A method to detect newborn nerve cells in regions with a low rate of neurogenesis. J. Neurosci. Methods.

[268] Leal-galicia P., Chávez-hernández M.E., Mata F., Mata-luévanos J., Rodríguez-serrano L.M., Tapia-de-jesús A., Buenrostro-jáuregui M.H. (2021). Adult neurogenesis: A story ranging from controversial new neurogenic areas and human adult neurogenesis to molecular regulation. Int. J. Mol. Sci..

[269] Luzzati F., de Marchis S., Parlato R., Gribaudo S., Schütz G., Fasolo A., Peretto P. (2011). New striatal neurons in a mouse model of progressive striatal degeneration are generated in both the subventricular zone and the striatal parenchyma. PLoS ONE.

